# Caveolin-1 in atherosclerosis: from endothelial lipoprotein transport to vascular immunometabolic remodeling

**DOI:** 10.3389/fcvm.2026.1954425

**Published:** 2026-09-24

**Authors:** Hongliang Yan, Jianying Lv, Jiali Bai, Yuci Wang, Chenxi Meng, Jiaxue Zhang, Jinlin Qin, Xuejun Li, Xinyu Zhang, Xiaguang Duan, Yude Zhu

**Affiliations:** 1 Department of Interventional neurology, Sionpharm North Hospital, Baotou, China; 2 Department of Anesthesiology, Inner Mongolia Baogang Hospital, Baotou, China

**Keywords:** atherosclerosis, caveolae, caveolin-1, endothelium, immunometabolism, low-density lipoprotein, mechanotransduction, transcytosis

## Abstract

Atherosclerosis is initiated by endothelial dysfunction and pathological retention of apolipoprotein B-containing lipoproteins within the arterial intima, yet the molecular mechanisms governing the earliest steps of lipoprotein entry remain incompletely understood. Caveolin-1 (Cav-1), the principal structural component of endothelial caveolae, has emerged as a critical regulator of vascular lipid transport, mechanotransduction, and inflammatory remodeling. However, its diverse and sometimes opposing biological functions have prevented a unified understanding of its role in atherosclerosis. In this review, we integrate recent advances regarding Cav-1-mediated regulation of atherogenesis, focusing on the transition from endothelial lipoprotein transport to vascular immunometabolic remodeling. We first discuss the structural and biochemical properties of caveolae and the scaffolding functions of Cav-1 that organize signaling and transport platforms. We then examine how Cav-1-dependent caveolar transcytosis facilitates receptor-guided low-density lipoprotein (LDL) transport through scavenger receptor class B type 1 (SR-B1), activin receptor-like kinase 1 (ALK1), and the DOCK4–RAC1 trafficking pathway, thereby controlling early lipid deposition in the arterial wall. Furthermore, we summarize how disturbed flow, glycocalyx disruption, and impaired autophagic turnover regulate Cav-1 abundance and localization, linking vascular biomechanics to lipid handling capacity. Importantly, we highlight the cell type- and disease stage-dependent effects of Cav-1. While endothelial Cav-1 promotes atherogenesis by enabling LDL transcytosis, macrophage Cav-1 deficiency may enhance Toll-like receptor 4/nuclear factor-*κ*B/NLRP3 inflammasome activation and inflammatory plaque remodeling. These opposing functions explain the limitations of systemic Cav-1 targeting and emphasize the need for compartment-selective therapeutic strategies. We then assess the human evidence, comprising CAV1 variant associations with coronary artery disease, plaque and circulating Cav-1 measurements, and monogenic loss-of-function syndromes, and we ask whether established cardiovascular drugs, including statins, PCSK9 inhibitors, and SGLT2 inhibitors, modify this axis. We then set out concrete delivery strategies, including ligand-directed nanocarriers and compartment-specific payload design, through which selective modulation could be achieved. By proposing Cav-1 as a molecular interface connecting mechanical stress, lipid trafficking, and immune-metabolic regulation, this review provides a comprehensive framework for understanding atherosclerosis pathogenesis and identifies potential therapeutic opportunities beyond conventional lipid-lowering approaches.

## 1 Introduction

Atherosclerosis begins at the endothelium, not in the plasma (1). That distinction matters more than it first appears. Circulating low-density lipoprotein (LDL) concentration predicts risk across populations with a consistency few biomarkers achieve, and lowering it lowers events (2). Yet plasma concentration alone does not explain where lesions form. They appear at the inner curvature of the aortic arch and at the carotid bifurcation. Adjacent straight segments stay clean for a lifetime, although they are bathed in exactly the same lipoprotein pool (3). Something local decides where the particle crosses. Understanding that decision is the point of this review.

The geometry of the problem is unforgiving. Endothelial junctions in healthy artery admit water, ions, and small solutes through gaps on the order of a few nanometres (4). An LDL particle measures roughly twenty to fifty nanometres across (5). Paracellular leakage therefore cannot account for the flux observed in intact vessels. The particle must be carried through the cell rather than between cells. The vehicle for that journey is the caveola, a flask-shaped invagination of fifty to one hundred nanometres. It forms on the luminal face, pinches off, traverses the cytoplasm, and fuses with the abluminal membrane. Caveolin-1 (Cav-1), a membrane protein of twenty-one to twenty-four kilodaltons (4), is the defining component of that structure and is expressed at high levels in endothelial cells.

What makes Cav-1 interesting is that it never did only one job. Early work framed it as an inhibitory scaffold that sequesters endothelial nitric oxide synthase (eNOS) and holds nitric oxide output low (5). Subsequent studies established it as a requirement for caveolae assembly and therefore for caveolae-dependent lipoprotein uptake and transport (6). More recent work positions the caveola as a mechanosensor whose surface density and subcellular distribution shift with the flow regime, and as a checkpoint that modulates innate immune signalling in macrophages (7). Each of these literatures is internally coherent. Read together they describe a protein that translates the physical and lipid environment of the membrane into the inflammatory and metabolic behaviour of the vessel wall.

Our aim is to build that integrated account rather than to catalogue findings. We organise the review around the trajectory of the lipoprotein particle, because that trajectory imposes a natural logic: architecture determines transport, transport determines retention and modification, retention determines the immune and metabolic response, and that response feeds back on the endothelium and alters transport again. Where the evidence is strong we say so plainly. Where it conflicts, and it conflicts more often than the reviews usually admit, we set the conflicting studies side by side and ask what experimental difference produced the discrepancy.

Three tensions run through the field and deserve flagging at the outset. First, atheroprotection in mice lacking Cav-1 was originally attributed to eNOS activation. Triple-knockout experiments removing eNOS, Cav-1, and the LDL receptor together show that protection persists without nitric oxide (8). The mechanism therefore lies in transport and inflammation rather than in nitric oxide signalling. Second, the timing question is unresolved and consequential: endothelial deletion of Cav-1 restrains lesion initiation, yet the same manoeuvre applied to established lesions fails to halt progression, implying that caveolae-dependent entry dominates early and yields to other routes later (8, 9). Third, essentially all causal data derive from mouse models, while human evidence remains correlative. A therapeutic proposal built on this foundation must be stated with corresponding restraint. The overall organisation of the pathway, from caveola assembly at the luminal membrane to immunometabolic remodelling of the intima, is summarised in Figure 1.

**Figure 1:**
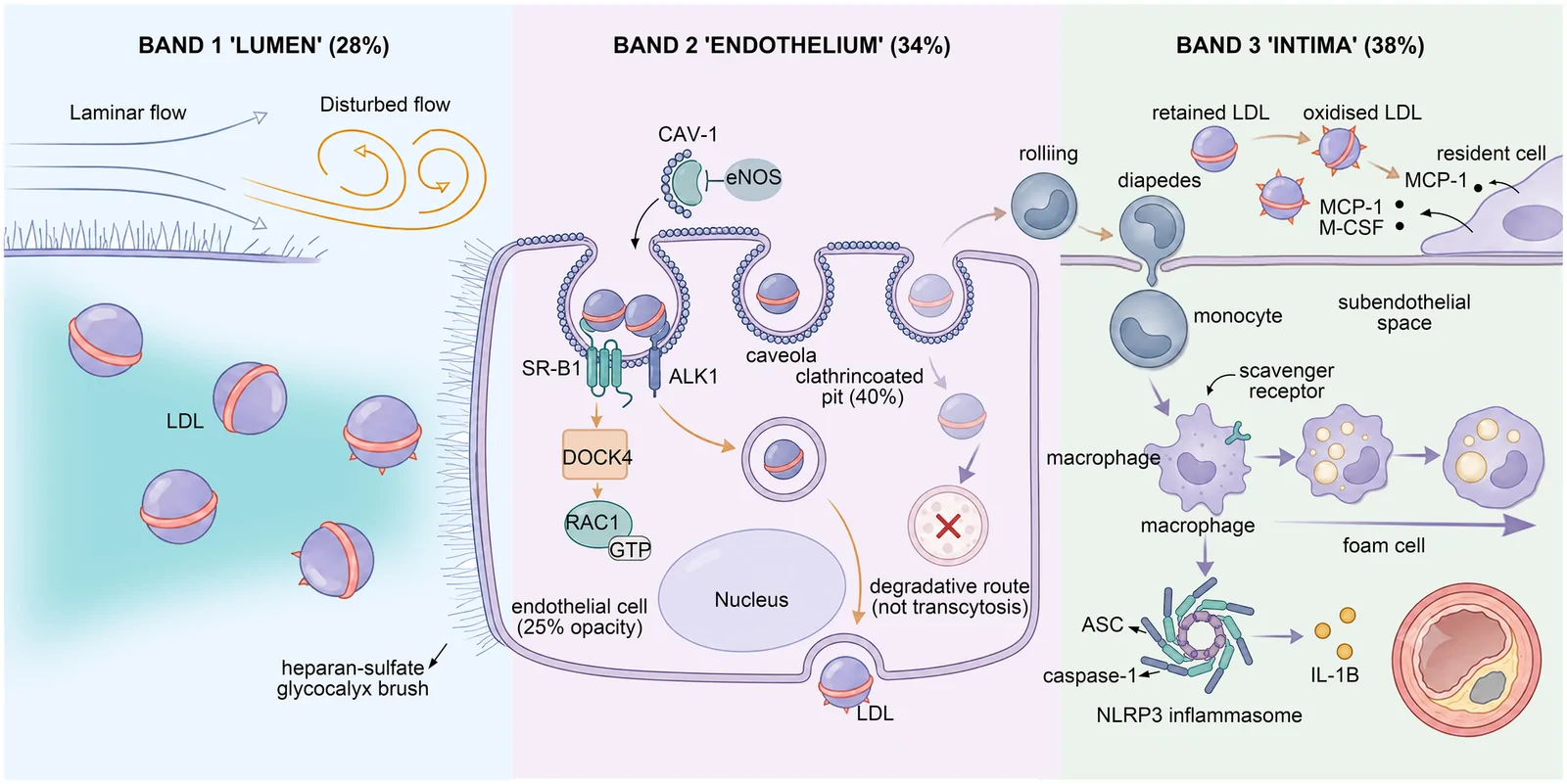
Overview of the caveolin-1 axis in atherogenesis, from luminal capture to intimal remodelling. Schematic overview of the pathway developed in this review. Circulating low-density lipoprotein encounters arterial endothelium, where caveolin-1-dependent caveolae capture the particle through scavenger receptor class B type 1 and activin receptor-like kinase 1 and carry it intact across the cell. Released lipoprotein is retained and oxidised in the subendothelial space, where it drives chemokine production, monocyte recruitment, macrophage differentiation, and foam cell formation, and ultimately NLRP3-dependent interleukin-1 beta release and necrotic core development. Disturbed flow and glycocalyx loss increase caveolin-1 availability and impair nitric oxide signalling, while reduced autophagic turnover raises steady-state caveolin-1 abundance. The panel establishes the section structure of the review, with numbered anchors indicating where each step is treated in detail.

The review proceeds in six movements. Section 2 covers caveolar architecture and the biochemical grammar of Cav-1 scaffolding. Section 3 follows LDL across the endothelium and dissects the receptor logic that guides it. Section 4 evaluates the genetic and pharmacological evidence, with the stage-dependence problem given the space it deserves. Section 5 treats mechanotransduction, the glycocalyx, and autophagic turnover as upstream controllers of Cav-1 abundance. Section 6 moves beyond the endothelium to macrophage immunometabolism and smooth muscle remodelling. Section 7 assembles the network and evaluates therapeutic tractability, including nanoparticle strategies that exploit caveolar uptake rather than suppress it. We close with the specific experiments that would move the field from association to mechanism.

## 2 Caveolar architecture and the biochemistry of caveolin-1

### 2.1 The caveola as a specialised membrane domain

Caveolae are not generic vesicles (10). They are compositionally distinct domains enriched in cholesterol and sphingolipids, and they adopt a characteristic bulb shape. They occur in terminally differentiated cells, and endothelium, adipocytes, and muscle show the highest densities (11). In arterial endothelium they can occupy a substantial fraction of the plasma membrane, which is itself a clue: a cell that devotes that much surface to one structure is using it for something quantitatively important, and in endothelium that something is transport (12).

Cav-1 is the marker protein and the organising element (13). It inserts into the inner leaflet as a hairpin, leaving both termini cytoplasmic, and oligomerises into units that then assemble into the coat (14). Cavin-1 is the second structural requirement. Without it, Cav-1 remains in the membrane but the invagination does not form properly (15). That dissociation separates the scaffolding role of Cav-1 from the curvature-generating role of the complete coat. This distinction matters for interpretation, because manipulations that remove Cav-1 remove both functions at once and cannot, by themselves, tell us which one produced a given phenotype (16).

### 2.2 Scaffolding and the caveolin-scaffolding domain

The caveolin-scaffolding domain, a short stretch in the juxtamembrane region of the N-terminus, binds a consensus motif present in a large number of signalling proteins (17). Binding generally holds the partner in a low-activity conformation. The canonical example is eNOS. It associates with Cav-1 in caveolae and is inhibited by that association. Release, driven by calcium-calmodulin or by AKT-dependent phosphorylation at serine 1,177, permits nitric oxide production. Under disturbed flow the equilibrium shifts toward the inhibited state, and Cav-1 phosphorylation and binding to eNOS lead to enzyme deactivation, which supplies a direct route from an unfavourable haemodynamic environment to reduced nitric oxide bioavailability (18).

It is worth resisting the temptation to read the scaffolding domain as a simple off switch. The same domain also concentrates partners, and concentration is not inhibition. A receptor held in a caveola is positioned adjacent to its effectors and to the membrane machinery that will internalise it, so the net effect of Cav-1 binding depends on which partner and which downstream step one measures (19). In the transport pathway discussed in Section 3, caveolar localisation of scavenger receptor class B type 1 and activin receptor-like kinase 1 is enabling rather than restraining, which shows that the scaffolding function operates in both directions.

### 2.3 Phosphorylation, cholesterol, and the regulated state of the coat

Cav-1 is phosphorylated on tyrosine 14 by Src family kinases, and this modification is a recurring theme in transport control (20). Binding of albumin to Gp60 triggers transcytosis through phosphorylation of Src family tyrosine kinases and of caveolin-1, illustrating a general principle in which a luminal ligand licences fission through a kinase relay rather than through mass action (21). Whether every transcytotic cargo uses this relay is not settled, but the architecture is conserved enough that Src activity has become a plausible pharmacological handle.

Cholesterol content is the other regulated variable. Caveolae require cholesterol for their integrity (22), which is why sterol-binding agents such as filipin disassemble them, and why cholesterol loading and depletion alter caveolar number in a way that couples the lipid state of the cell to its transport capacity. This creates an obvious feed-forward possibility: a cholesterol-rich environment supports more caveolae, more caveolae support more lipoprotein entry, and more entry raises local cholesterol further. Whether that loop operates with meaningful gain *in vivo* is a question we return to in Section 7, since it bears directly on whether Cav-1 is a driver or a passenger during lesion growth. Domain organisation of caveolin-1 and the principal regulated interactions of the caveolar coat are depicted in Figure 2.

**Figure 2:**
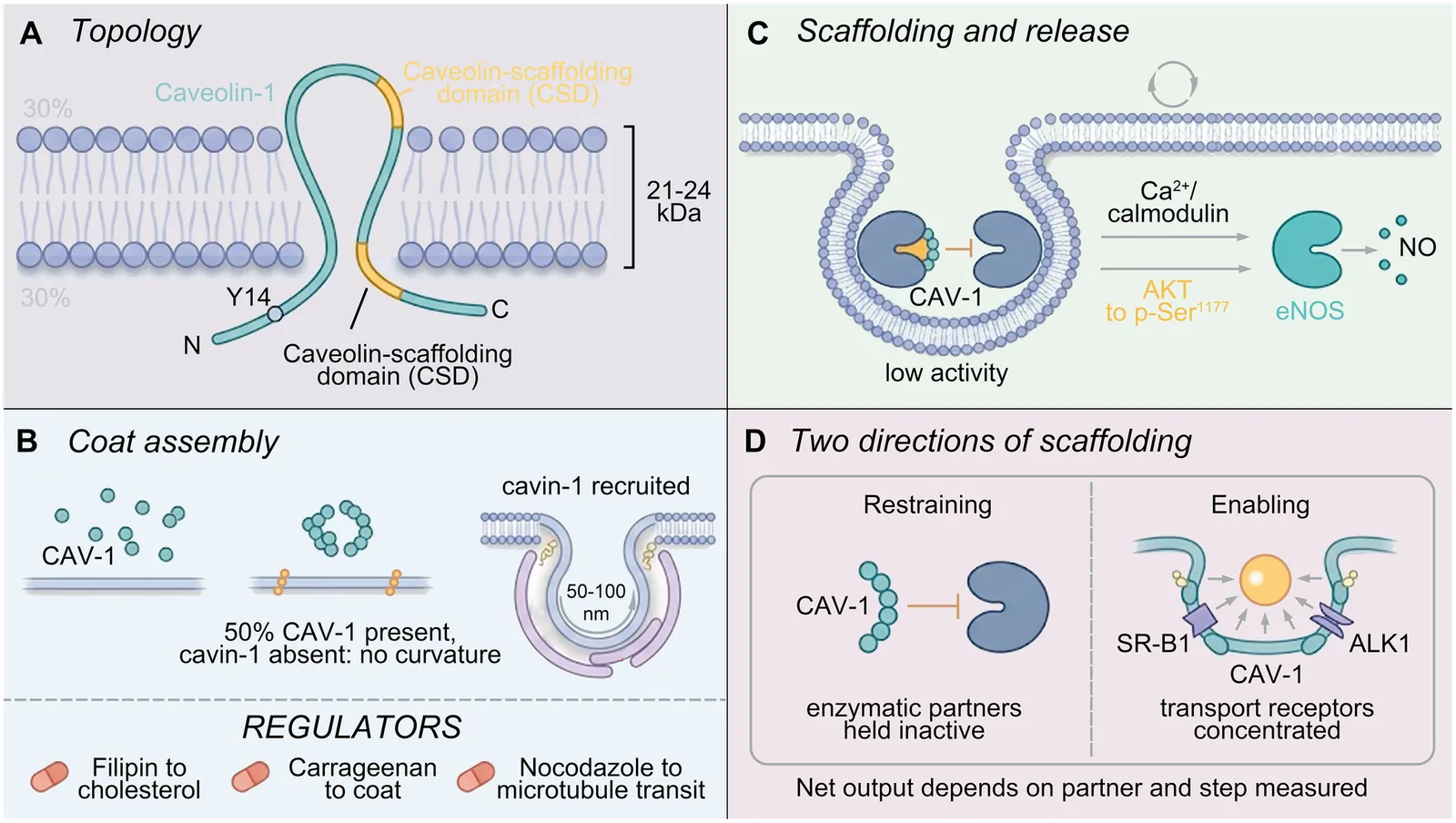
Domain organisation of caveolin-1 and the regulated state of the caveolar coat. Structural and biochemical features of caveolin-1. The protein adopts a hairpin topology within the inner leaflet, leaving both termini in the cytoplasm, and oligomerises to form the caveolar coat together with cavin-1. The caveolin-scaffolding domain binds a consensus motif shared by numerous partners and generally maintains them in a low-activity conformation, as illustrated for endothelial nitric oxide synthase, which is released upon calcium-calmodulin binding or AKT-dependent phosphorylation at serine 1,177. Phosphorylation of caveolin-1 at tyrosine 14 by Src family kinases licences vesicle scission. Cholesterol is required for coat integrity, and the points of action of filipin, carrageenan, and nocodazole are indicated. Panel D shows the dual and context-dependent consequence of scaffolding, which restrains enzymatic partners while concentrating transport receptors.

Taken together, the architecture supports a reading in which Cav-1 is less a switch than a hub whose output depends on context. That framing anticipates the contradictions catalogued later, since a hub embedded in several circuits will produce opposite phenotypes when different circuits dominate.

## 3 Endothelial lipoprotein transport: following the particle

### 3.1 Why transcytosis, and why it is the rate-limiting step

The subendothelial retention of apolipoprotein B-containing lipoproteins is the initiating event of atherogenesis, and everything downstream, including oxidative modification, monocyte recruitment, and foam cell formation, is conditional on that retention (22, 23). Since the particle cannot pass between cells in a healthy monolayer, the transcellular route is not one option among several but the only route available in the pre-lesional artery. This gives endothelial transcytosis an unusual position in the causal chain: it is both necessary and, in early disease, essentially sufficient to set the substrate load of the intima.

The distinction between transcytosis and classical receptor-mediated endocytosis is central and is frequently blurred. When LDL is internalised through the LDL receptor, the complex enters clathrin-coated pits. The acidic endosome then dissociates ligand from receptor. The receptor recycles and the particle proceeds to lysosomal degradation (24). That pathway consumes the particle. Transcytosis does the opposite: it preserves the particle intact and deposits it on the far side. The two pathways therefore have opposite consequences for the intima, even though both begin with LDL binding at the luminal surface. A treatment that shifted flux from one to the other would be atheroprotective without changing plasma lipids at all. This is the conceptual foundation for transport-selective therapy discussed in Section 7.

### 3.2 The receptor logic: SR-B1, ALK1, and the LDL receptor

Three receptors dominate the discussion, and their division of labour is now reasonably clear. Scavenger receptor class B type 1 (SR-B1) and activin receptor-like kinase 1 (ALK1) both localise to caveolae, both bind LDL directly, and both facilitate transport of the intact particle across the endothelial cell (25). The LDL receptor, by contrast, principally routes the particle to degradation. Evidence that degradation of the LDL receptor has no effect on transcytosis reinforces the separation, and it explains an otherwise puzzling observation, namely that the receptor most famous for LDL handling is not the receptor that delivers LDL into the wall (26).

Quantitatively, the contribution of these two receptors is large. Eliminating endothelial expression of SR-B1 or ALK1 reduces LDL transport *in vivo* by approximately half, and combined inhibition reduces it by roughly seventy per cent (27). Numbers of that magnitude are rare in vascular biology and they carry a clear implication: the receptor-guided arm of transcytosis, not bulk fluid-phase uptake, carries most of the flux in the mouse (26). The residual thirty per cent is not trivial either, and it is a reasonable inference that a fluid-phase component persists, since up to seven LDL particles can be close-packed within a single caveola and would be carried whether or not a receptor engaged them.

### 3.3 The SR-B1 to DOCK4 to RAC1 axis

The most completely resolved mechanism runs through SR-B1. LDL particles colocalise with SR-B1 in endothelial intracellular vesicles *in vivo* (28), and transcytosis across endothelial monolayers requires direct binding of LDL to SR-B1 together with an eight-amino-acid cytoplasmic domain of the receptor that recruits the guanine nucleotide exchange factor dedicator of cytokinesis 4 (DOCK4) (26). DOCK4 in turn promotes internalisation of SR-B1 and activates RAC1 (29), and this small GTPase step is what commits the vesicle to scission and transit. In mice, SR-B1 in endothelial cells mediates delivery of LDL into arteries and its accumulation by artery wall macrophages, thereby promoting atherosclerosis (30).

The elegance of this axis lies in its modularity. The ligand-binding function and the trafficking function reside in physically separate parts of the receptor, which means they are separately druggable. An agent that occludes the eight-residue cytoplasmic motif, or that blocks the DOCK4 interaction, would in principle abolish transcytosis while leaving the extracellular lipoprotein-binding activity of SR-B1 intact (31). That distinction is not academic, because SR-B1 has a well-established beneficial role in hepatic reverse cholesterol transport, and a systemic ligand-blocking drug would compromise it. Selectivity here is not a refinement; it is the difference between a viable and an unviable target. The receptor-guided transcytosis pathway, from luminal capture through DOCK4 and RAC1-dependent scission to abluminal release (29, 32, 33), is shown in Figure 3.

**Figure 3:**
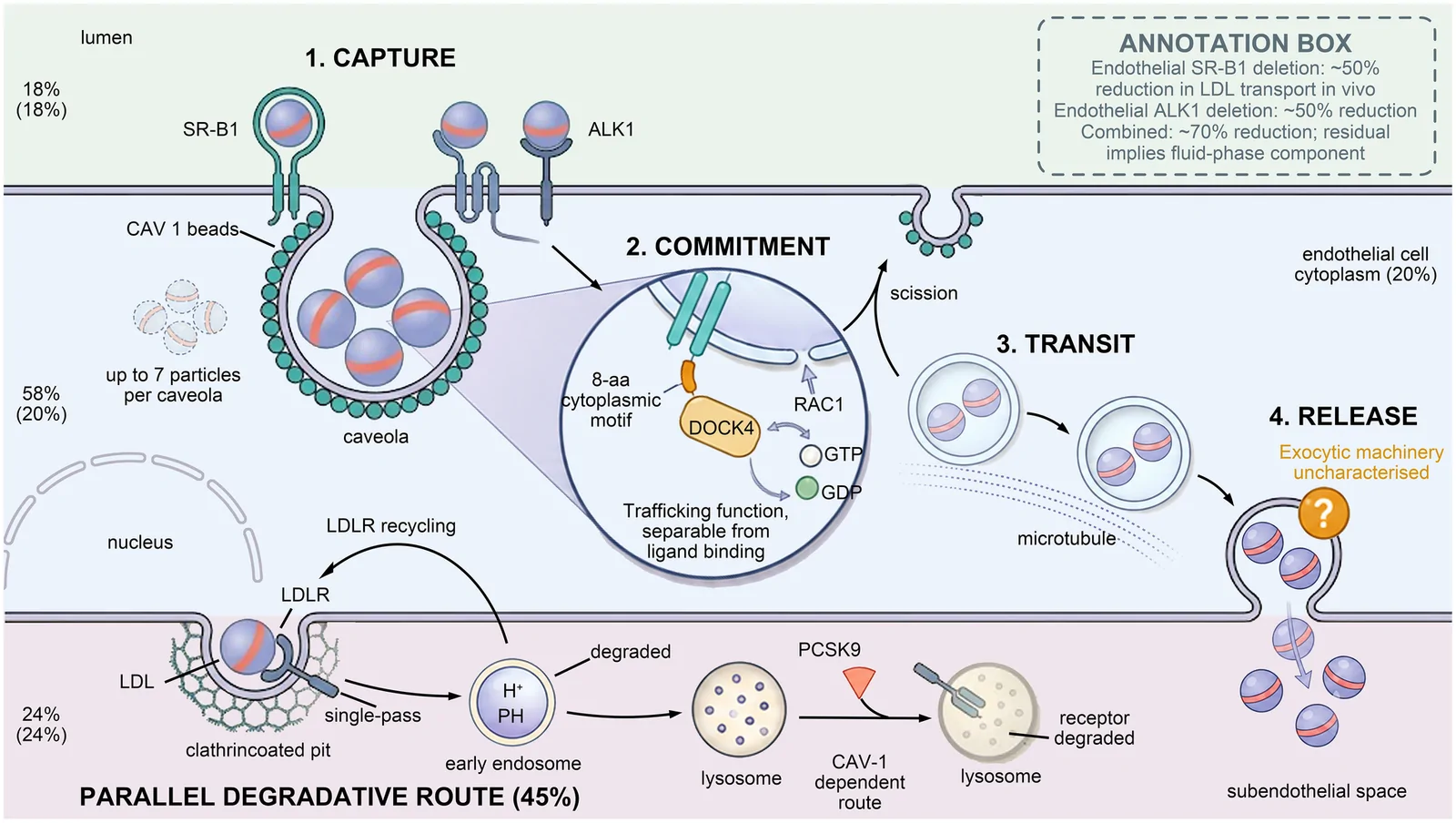
Receptor-guided transcytosis of low-density lipoprotein across arterial endothelium. The transcytotic route in molecular detail. Low-density lipoprotein binds scavenger receptor class B type 1 and activin receptor-like kinase 1 within the caveola. Binding to scavenger receptor class B type 1 exposes an eight-amino-acid cytoplasmic segment that recruits dedicator of cytokinesis 4, which promotes receptor internalisation and activates RAC1, thereby committing the vesicle to scission and transit. The particle crosses intact and is released by exocytosis, a step whose machinery remains uncharacterised and which is marked accordingly. The degradative route through the low-density lipoprotein receptor is shown in parallel for contrast, since it consumes rather than delivers the particle, and the diversion imposed by proprotein convertase subtilisin/kexin type 9 is indicated. Reported effect sizes for endothelial receptor deletion *in vivo* are annotated. Up to seven particles may be close-packed within a single caveola, which underlies the persisting fluid-phase component.

### 3.4 Cav-1 as the obligatory platform

Where does Cav-1 sit in this scheme? It is the platform on which the scheme runs. Cav-1 is required for the formation of caveolae and for caveolae-mediated LDL uptake and transcytosis across the endothelium, and since SR-B1 and ALK1 both reside within caveolae, loss of the coat displaces the receptors from the structure that would otherwise internalise them (34–36). The receptors increase transcytosis at least in part by concentrating LDL within the caveolae, which frames Cav-1 as the element that converts a binding event into a transport event.

This platform role also predicts an important asymmetry. Removing a receptor removes one guided input while leaving the vesicular machinery available to other inputs, whereas removing Cav-1 removes the machinery itself. One would therefore expect Cav-1 deletion to produce a larger and less specific effect than deletion of any single receptor, and to carry more collateral consequences. Both expectations are borne out, and the second is the reason Cav-1 has proven difficult to translate.

### 3.5 Oxidised LDL and the modification loop

Caveolae and Cav-1 also mediate endocytosis and transcytosis of oxidised LDL (37). Work in human umbilical vein endothelial monolayers using caveolae inhibitors demonstrates the point pharmacologically: carrageenan, filipin, and nocodazole reduce transport of oxidised LDL across the monolayer by roughly forty-nine, seventy-two, and eighty per cent respectively, a spread that reflects the different steps each agent blocks, from coat integrity to microtubule-dependent transit. The convergence of native and oxidised particles on the same structure is mechanistically tidy but pathologically unfortunate, because it means the pathway that delivers substrate also delivers the modified ligand that amplifies inflammation.

The sequence in the intima is well described. LDL that enters the subendothelial space undergoes mild oxidation by resident vascular cells, which then stimulates production of cytokines including monocyte chemoattractant protein 1 and macrophage colony-stimulating factor (38). These promote recruitment of monocytes and their differentiation into macrophages. The macrophages further oxidise LDL through myeloperoxidase and reactive oxygen species. Macrophage scavenger receptors then recognise and internalise the oxidised particles, and foam cells form (39). What began as a transport problem has become an immune problem, and the transition is where Sections 5 and 6 pick up.

## 4 Genetic and pharmacological evidence: what the models actually show

### 4.1 The foundational knockout phenotype

The genetic case begins with a clean result. Genetic loss of Cav-1 reduces LDL infiltration into the arterial wall, promotes nitric oxide production, and lowers expression of adhesion molecules. Endothelial-specific Cav1 transgene expression reverses all of these effects (40). The rescue is what elevates this from correlation to causation, and it also localises the relevant pool of Cav-1 to the endothelium rather than to smooth muscle or macrophages (40). Complementary work confirms that re-expression of caveolin-1 in endothelium rescues the vascular, cardiac, and pulmonary defects of global caveolin-1 knockout mice, which is a useful reminder that the global knockout is a systemically abnormal animal and that its vascular phenotype should not be read in isolation.

### 4.2 Uncoupling nitric oxide from atheroprotection

The original interpretation was that absence of Cav-1 releases eNOS, raises nitric oxide, and protects by that route (41). It was a reasonable inference and it turned out to be incomplete. Triple-knockout mice lacking eNOS, Cav-1, and the LDL receptor were generated to test whether nitric oxide production is required for the protection. It is not. Cav-1 and caveolae act instead as a central regulator that links biomechanical, metabolic, and inflammatory pathways, independent of endothelial eNOS activation and nitric oxide production (42). The same work attributes the protection to attenuated LDL transcytosis and reduced vascular inflammation, with effects on extracellular matrix remodelling and fibronectin deposition contributing as well.

This result deserves more weight than it usually receives, because it does two things at once. It removes the tidiest available explanation, and it redirects attention to transport, which is precisely the function that later receptor work has resolved in molecular detail. The two literatures were developed largely independently and they converge, which is the kind of convergence that should increase confidence.

### 4.3 The stage-dependence problem

Here the picture complicates, and honesty requires that the complication be placed in the foreground rather than in a limitations paragraph. Progression of established atherosclerotic lesions is not inhibited by endothelial knockout of caveolin-1 (43). In normal arteries and early lesions, transcytosis through endothelial caveolae may represent the primary route of LDL entry, but this pathway appears to become less dominant in established disease, and murine atherosclerotic lesions exhibit a high capacity for LDL entry and retention through additional routes (44).

The implications are substantial. If caveolar transcytosis governs initiation but not progression, then Cav-1 is a prevention target rather than a treatment target, and the clinical population in which it would be tested is the population least likely to accept a novel vascular intervention (45). It also implies that the endothelium of an established lesion is a different tissue from the endothelium of a normal artery, with junctional integrity compromised, neovascularisation supplying alternative entry, and retention within the matrix dominating over entry rate as the determinant of accumulation (46). Any therapeutic programme built on Cav-1 must confront this directly, and we take it up again in Section 7.

### 4.4 Reconciling divergent reports

The Cav-1 literature contains genuine disagreements, and most of them dissolve on inspection into differences of cell type, model, or timing rather than into irreconcilable biology. Table 1 assembles the principal studies for direct comparison. Three sources of divergence recur and are worth naming explicitly.

**Table 1:** Genetic, pharmacological, and mechanistic studies of caveolin-1 and caveolae-dependent lipoprotein transport in atherosclerosis, arranged by mechanistic theme.

#	Study focus	Model or system	Compartment	Principal finding relevant to this review	Authors
1	Endothelial Cav-1 and atherogenesis	Cav1-null and endothelial Cav1 transgenic mice	Endothelium	Endothelial Cav-1 drives LDL infiltration into the arterial wall. An endothelial transgene reverses every effect of deletion, localising the causal pool to endothelium.	Takeshi Kanda, et al. (48)
2	Cav-1, transcytosis, and inflammation independent of eNOS	eNOS/Cav1/Ldlr triple-knockout mice	Endothelium	Atheroprotection from Cav-1 loss does not require nitric oxide. Transport, inflammation, and matrix remodelling account for it.	Cristina M Ramírez, et al. (49)
3	Stage-dependence of endothelial Cav-1 deletion	Endothelial Cav1 knockout, established lesions	Endothelium	Endothelial Cav-1 deletion does not slow established lesions. Caveolar entry dominates in normal artery and early lesion only.	Rocío Muñiz-Anquela, et al. (43)
4	SR-B1 to DOCK4 to RAC1 axis	Endothelial-specific Scarb1 deletion; monolayer transcytosis	Endothelium	SR-B1 delivers LDL into the artery wall and into wall macrophages. Transcytosis requires LDL binding plus an eight-amino-acid cytoplasmic motif that recruits DOCK4 and activates RAC1.	Linzhang Huang, et al. (31)
5	Quantitative receptor contribution	Murine *in vivo* LDL transport	Endothelium	Deleting endothelial SR-B1 or ALK1 halves LDL transport *in vivo*. Combined inhibition reduces it by about 70%, and the residual implies a fluid-phase component.	Israel O Bolanle, et al. (26)
6	Caveolae, ALK1, SR-B1 as transport regulators	Review of transport mechanisms	Endothelium	Cav-1 is required for caveolae formation and for caveolar LDL transcytosis. SR-B1 and ALK1 bind LDL directly and are candidate therapeutic targets.	Chi Zhang, et al. (34)
7	Cav-1-mediated LDL transcytosis	Comprehensive mechanistic review	Endothelium	Cav-1 is indispensable for LDL transcytosis. LDL binding to SR-B1 recruits DOCK4 and activates RAC1. The exocytic release step remains undefined.	Yifei Zhang, et al. (50)
8	Cav-1 and the fate of LDL in endothelium	Mechanistic review	Endothelium	LDLR-internalised LDL is degraded and the receptor recycles. PCSK9 engagement diverts the complex through Cav-1 and the receptor is degraded.	Alessandra Puddu, et al. (51)
9	Oxidised LDL transcytosis through caveolae	HUVEC monolayer on collagen-coated filter	Endothelium	Caveolae inhibitors block oxidised LDL transport across endothelial monolayers. Carrageenan, filipin, and nocodazole reduce it by 48.9%, 72.4%, and 79.8%.	Shao-wei Sun, et al. (52)
10	Endothelial Cav-1 as therapeutic target	Mechanistic review	Endothelium	Nutrient depletion activates mTORC1 and inhibits AMPK. ULK1 and class III PI3K complexes fail to form, autophagic degradation of Cav-1 falls, and transcytosis rises.	Yan Shu, et al. (53)
11	Glycocalyx control of caveolar mechanosignalling	Endothelial cells under uniform vs. disturbed flow; heparinase III	Endothelium	Disturbed flow lowers Cav-1 and abolishes its positional preference. Heparan sulfate removal reproduces the disturbed-flow phenotype under uniform flow, placing the glycocalyx upstream.	Harding IC, et al. (54)
12	Caveolae as flow-sensitive domains	Endothelial mechanotransduction review	Endothelium	Caveolae redistribute with shear and concentrate upstream under uniform flow. Disturbed flow removes the positional preference and drives Cav-1 phosphorylation and eNOS deactivation.	Mohammad Hamrangsekachaee, et al. (55)
13	Cav-1 and caveolae in mechanotransduction	Murine vessels, direct mechanotransduction evidence	Endothelium/vessel wall	Cav-1 and caveolae mediate mechanotransduction in intact vessels. They contribute directly to vascular remodelling.	Chi Zhang, et al. (34)
14	Endothelial re-expression rescue	Global Cav1-null mice with endothelial re-expression	Endothelium	Endothelial re-expression rescues the vascular, cardiac, and pulmonary defects of the global knockout. The global model is systemically abnormal and must be read with caution.	Takahisa Murata, et al. (56)
15	Endothelial CB1 and caveolar transport	Endothelial Cnr1 knockout mice	Endothelium	Endothelial Cnr1 deletion downregulates CAV1 and ALK1 and reduces apical caveolae. LDLR, SR-B1, and LOX-1 are unaffected, and effects are more pronounced in females.	Bingni Chen, et al. (57)
16	Salidroside and autophagic targeting of Cav-1	Endothelial cells, LDL transcytosis assay	Endothelium	Autophagic targeting of active Src and Cav-1 suppresses LDL transcytosis. Cav-1 abundance is therefore pharmacologically addressable.	Rong Wang, et al. (58)
17	Vascular bed specificity of LDL handling	Brain vs. aortic endothelial cells	Endothelium	Brain endothelium degrades rather than transports LDL through LDLR and ALK1. Identical receptors produce opposite outcomes in different vascular beds.	Sofia Kakava, et al. (27)
18	Caveolar transcytosis at the blood-brain barrier	Murine central nervous system endothelium	Endothelium	Lipid transport-dependent suppression of caveolar transcytosis maintains blood-brain barrier integrity. An endogenous brake on the caveolar route exists.	Kunjian Yang, et al. (37)
19	LOX-1 as endothelial oxidised LDL receptor	Bovine aortic endothelial cells	Endothelium	LOX-1 is an endothelial receptor for oxidised LDL. Modified particles have a receptor route distinct from native transcytosis.	Israel O Bolanle, et al. (26)
20	Cav-1 and TLR regulation in macrophages	Murine macrophages, Cav-1 deletion	Macrophage	Cav-1 interacts with multiple Toll-like receptors to regulate phagocytosis and activation. Its deletion increases pro-inflammatory output, inverting the endothelial sign.	Ayyanar Sivanantham, et al. (59)
21	Cav-1 to TLR4 to NLRP3 axis	Mechanistic review	Macrophage	TLR4 and NF-kB signalling drives NLRP3 transcription. Cav-1 loss augments inflammasome priming and plaque destabilisation.	Xuan Liu, et al. (40)
22	Oxidised LDL and NLRP3-dependent pyroptosis	Wild-type and Nur77-null peritoneal macrophages	Macrophage	Oxidised LDL triggers NLRP3-dependent pyroptosis in macrophages. Lactate dehydrogenase release rises with cleaved caspase-1, cleaved IL-1B, and gasdermin D.	Ruosen Yuan, et al. (60)
23	NLRP3 structure and two-signal activation	Mechanistic review	Macrophage/immune	NLRP3 activation follows two-signal logic. Priming through TLR and NF-kB drives transcription; triggering drives oligomerisation, caspase-1 activation, and pyroptosis.	Na Lu, et al. (61)
24	IL-1B neutralisation in human disease	Randomised controlled trial, CANTOS	Clinical	IL-1B neutralisation reduces cardiovascular events in a randomised trial. This is the strongest human validation of the inflammasome arm.	Jie Tan, et al. (62)
25	Chaperone-mediated autophagy and inflammasome	Murine atherosclerosis progression	Macrophage/intima	Chaperone-mediated autophagy becomes impaired as lesions progress. NLRP3 activation and IL-1B secretion rise from the same upstream defect that raises Cav-1.	Lei Qiao, et al. (63)
26	Autophagy proteins and endothelial integrity	ATG16L1 and ATG5 deficiency models	Endothelium/macrophage	ATG16L1 and ATG5 deficiency raises IL-1B and PECAM-1 and disrupts endothelial contact structures. Neutrophil penetration and endothelial dysfunction follow.	Natalia Reglero-Real, et al. (64)
27	ATG14 and macrophage lipid degradation	ATG14 overexpression models	Macrophage	ATG14 overexpression promotes autophagosome-lysosome fusion through syntaxin 17. Macrophage lipid degradation increases, apoptosis falls, and disease slows.	Hui Zhang, et al. (65)
28	NLRP3 siRNA nanocarrier targeting inflamed endothelium	VCAM-1 peptide-targeted cationic liposomes, *in vitro* and *in vivo*	Endothelium	VCAM-1 peptide-targeted liposomes deliver NLRP3 siRNA to inflamed endothelium. Inflammasome activation and Cav-1-mediated LDL transcytosis are both blocked.	Yifei Zhang, et al. (50)
29	Endothelial Cav-1 in lesion development	Murine atherosclerosis	Endothelium	Endothelial Cav-1 contributes substantially to lesion development. The result supports the transport-centred interpretation.	Stephanos Pavlides, et al. (66)
30	Piezo1 mechanotransduction under disturbed flow	HUVEC and murine models	Endothelium	Disturbed flow drives endothelial inflammation through Piezo1, CaMKII, FAK/Src, and YAP. Caveolae are one mechanosensor among several.	Yining Lan, et al. (67)

Studies are grouped by mechanistic theme rather than chronology: transport and receptor logic (entries 1 to 9), upstream mechanical and metabolic control (entries 10 to 19), immunometabolic output (entries 20 to 27), and therapeutic or integrative evidence (entries 28 to 30). Compartment denotes the cellular pool to which the principal finding is attributed, which is the variable most frequently confounded in global deletion models. Percentage values are reported as stated in the source. Where a study is cited for more than one distinct mechanistic claim it appears once per claim, with the compartment column disambiguating. Identifiers are given as PMID where available, otherwise PMCID or DOI. ALK1, activin receptor-like kinase 1; ASC, apoptosis-associated speck-like protein containing a caspase recruitment domain; CAMKII, calcium/calmodulin-dependent protein kinase II; Cav-1, caveolin-1; DOCK4, dedicator of cytokinesis 4; eNOS, endothelial nitric oxide synthase; HUVEC, human umbilical vein endothelial cell; IL-1B, interleukin-1 beta; LDL, low-density lipoprotein; LDLR, low-density lipoprotein receptor; LOX-1, lectin-like oxidised LDL receptor 1; NF-kB, nuclear factor kappa B; NLRP3, NOD-like receptor family pyrin domain containing 3; PCSK9, proprotein convertase subtilisin/kexin type 9; PECAM-1, platelet endothelial cell adhesion molecule 1; SR-B1, scavenger receptor class B type 1; TLR, Toll-like receptor; TNF, tumour necrosis factor; VCAM-1, vascular cell adhesion molecule 1; YAP, yes-associated protein.

The first is compartment. Global deletion removes Cav-1 from endothelium, smooth muscle, macrophages, adipocytes, and fibroblasts simultaneously, and these pools do not act in the same direction (34). Endothelial Cav-1 promotes lesion formation by enabling transport, whereas loss of Cav-1 has been associated with enhanced Toll-like receptor 4 and nuclear factor kappa B activity in macrophages (47), which would augment priming of the NLRP3 inflammasome and act in the opposite direction. A global knockout reports the sum of these vectors, and the sum is not informative about any of them.

The second is stage, discussed above. The third is sex and hormonal state, which is under-examined. Transcytosis of LDL through caveolae is inhibited by oestrogen, which may contribute to the protective effect against early atherosclerosis in females, and recent work on endothelial cannabinoid receptor 1 found effects on vascular lipid transport that were more pronounced in females. Studies that pool sexes may therefore be averaging across a real biological difference in the very pathway under study. Genetic and pharmacological studies of caveolin-1 and caveolar transport in atherosclerosis, together with model, compartment, and principal outcome, are compiled in Table 1.

### 4.5 Pharmacological probes and their limits

Pharmacological disruption of caveolae has been informative and is uniformly non-selective (68). Filipin sequesters cholesterol, carrageenan and nocodazole act on coat assembly and microtubules respectively, and all three suppress lipoprotein transport across endothelial monolayers, but none is remotely usable *in vivo* (26). Their value is as mechanistic probes, not as leads.

More interesting are agents that act on the regulatory layer rather than on the structure. Salidroside suppresses LDL transcytosis across endothelial cells through autophagic targeting of active Src and caveolin-1, which is notable because it exploits an endogenous degradative route rather than a chemical one and therefore has a plausible therapeutic logic (26). Whether the effect size and selectivity survive translation is unknown, and the compound has not been tested in the way that would answer the question. It nonetheless establishes proof of principle that Cav-1 abundance is pharmacologically addressable through autophagy, which connects directly to the mechanistic material in Section 5.

### 4.6 Human genetic, tissue, and biomarker evidence

Everything above rests on mouse genetics, and a reader is entitled to ask what the human data show. Three classes of human evidence exist: common genetic variation at the CAV1 locus, measurements of Cav-1 protein in plaque and in plasma, and monogenic loss-of-function syndromes. None is causal in the way a conditional knockout is. Taken together they nonetheless constrain which parts of the murine account can be carried into human disease, and they do so unevenly. Table 2 assembles them.

**Table 2:** Human genetic, tissue, and circulating-biomarker evidence relating caveolin-1 to atherosclerotic cardiovascular disease.

#	Evidence class	Population or specimen	Principal finding	Compartment interpretation	Source
1	Common variant, rs3807989 (*CAV1*/*CAV2*)	3,299 cases and 2,886 controls, three Chinese Han populations	The minor A allele is associated with coronary artery disease and myocardial infarction (OR 1.19; adjusted *P* = 2.18 × 10^−5^). The risk genotype raises *CAV1* expression.	Directionally concordant with the endothelial transport arm	Shanshan Chen, et al. (69)
2	Common variant, metabolic syndrome	Caucasian and Hispanic cohorts	A prevalent *CAV1* variant is associated with the metabolic syndrome.	Systemic and compartment-blind	Rene Baudrand, et al. (70)
3	Plaque protein abundance	378 carotid endarterectomy specimens	Plaque Cav-1 is lower than in non-atherosclerotic artery. Low Cav-1 tracks with lipid core, macrophage infiltration, and MMP-9 activity.	Reports the macrophage and matrix arm, not endothelium	Juan A Rodriguez-Feo, et al. (71)
4	Plaque protein abundance	Human atheroma	Cav-1 is decreased in atheroma with loss of antiproliferative control of smooth muscle cells.	Late-stage, non-endothelial	Carsten Schwencke, et al. (72)
5	Circulating biomarker	160 adults undergoing coronary angiography	Serum Cav-1 is lower in coronary artery disease and inversely associated with risk after adjustment.	Compartment of origin unknown; biomarker association only	Jing Chen, et al. (73)
6	Monogenic loss of function	Human kindreds, pulmonary arterial hypertension	Loss-of-function *CAV1* variants segregate with heritable pulmonary arterial hypertension.	Systemic Cav-1 loss is not benign	Eric D Austin, et al. (74)
7	Monogenic loss of function	Congenital generalised lipodystrophy	A homozygous nonsense *CAV1* mutation causes generalised lipodystrophy with insulin resistance and hypertriglyceridaemia.	Constrains systemic suppression strategies	Chong A Kim, et al. (75)
8	Human tissue mechanism	Cultured arteries and macrophages, scaffolding-domain peptide	A peptide mimicking the caveolin-scaffolding domain lowers gelatinase and MMP-9 activity.	Supports protease restraint by Cav-1 in the plaque compartment	Juan A Rodriguez-Feo, et al. (71)

Evidence class denotes the type of human observation rather than the strength of inference. Compartment interpretation is our reading of which cellular pool the observation most plausibly reflects, and it is offered explicitly as interpretation. No entry in this table establishes causation. Odds ratios are reported as stated in the source. Abbreviations are as defined in Table 1.

The strongest signal comes from common variation. The tagSNP rs3807989 in intron 2 of CAV1 was tested in three independent Chinese Han populations, 3,299 coronary artery disease cases and 2,886 controls in total. The minor A allele was associated with disease in each population and in the combined analysis (adjusted *P* = 2.18 × 10^−5^, odds ratio 1.19), the association extended to myocardial infarction, and the risk genotype was associated with higher expression of CAV1 but not of CAV2 (69). The direction of effect is what matters here. More Cav-1 associates with more disease in humans, which is the same direction as the endothelial transport arm of this review. This is the only human genetic observation that is directionally concordant with the murine transcytosis data, and it is therefore the single most informative human result we are aware of. Its limits should be stated with equal clarity: the cohorts are Chinese Han, replication across ancestries is incomplete, variants at this locus have also been associated with atrial fibrillation and with metabolic traits, and a germline variant alters Cav-1 in every tissue rather than in endothelium alone.

That last point is the compartment problem reappearing in human genetics. A prevalent CAV1 variant is associated with the metabolic syndrome in Caucasian and Hispanic cohorts (70), and CAV1 genotype modifies the cardiometabolic response to dietary fat. These are systemic phenotypes. A germline variant is, in effect, a lifelong global knockdown or knock-up, and it confounds endothelial, macrophage, adipocyte, and hepatic contributions exactly as the global mouse knockout does. Human genetics therefore tells us that the gene matters; it does not tell us which compartment carries the effect.

Protein measurements in human tissue point the other way, and the discrepancy is instructive rather than fatal. In 378 carotid endarterectomy specimens, Cav-1 was lower in plaque than in non-atherosclerotic arterial tissue. Low plaque Cav-1 tracked with features of instability, including a large lipid core, thrombus, macrophage infiltration, raised interleukin-6 and interleukin-8, and elevated MMP-9 activity, and it was more pronounced in men and in patients with previous myocardial infarction. Plaque Cav-1 above the median was associated with the absence of new vascular events in the first thirty days after surgery (71). An independent series likewise reported decreased Cav-1 in atheroma with loss of antiproliferative control over smooth muscle cells (72). Read naively this reverses the mouse result. Read against Section 6 it does not. Advanced plaque tissue is dominated by macrophages and modulated smooth muscle rather than by intact endothelium, and in those compartments Cav-1 restrains protease activity and inflammatory output. The human tissue data are therefore a readout of the macrophage and matrix arm, while the genetic data are a readout of the lifelong endothelial arm. The sign inversion argued in Section 6.1 predicts precisely this pattern.

Circulating Cav-1 has recently been examined as a biomarker. In a retrospective cohort of 160 Chinese adults undergoing coronary angiography, serum Cav-1 was lower in patients with angiographic disease and showed an independent, approximately linear inverse association with disease risk after adjustment (73). The sample is small, the design retrospective and single-centre, the tissue of origin of circulating Cav-1 unknown, and reverse causation unexcluded. We regard this as hypothesis-generating for risk stratification and as uninformative about endothelial transport, since a plasma concentration cannot distinguish the compartment from which the protein was shed.

Monogenic human disease supplies a different kind of constraint. Loss-of-function variants in CAV1 cause heritable pulmonary arterial hypertension (74) and congenital generalised lipodystrophy with severe insulin resistance and hypertriglyceridaemia (75). Whatever else these syndromes show, they establish that profound Cav-1 deficiency in humans is not benign. Any proposal to lower Cav-1 systemically inherits that observation, which is an additional and independent argument for the compartment-selective strategies discussed in Section 7.5.

What would settle the human question is absent from the literature. No Mendelian randomisation analysis has been performed using an instrument specific to endothelial CAV1 expression with lipoprotein retention as the outcome. No human study relates regional caveolar density to regional lipoprotein retention within the same artery, although carotid endarterectomy specimens make this feasible. And no agent selective enough for a trial exists. Until those gaps are closed, the human layer constrains the murine account without confirming it, and the therapeutic statements in Section 7 should be read with that qualification in force.

## 5 Upstream control: mechanotransduction, the glycocalyx, and autophagic turnover

### 5.1 Flow patterns set caveolar density and position

Lesions form where flow is disturbed, and caveolae are among the structures that register the difference (76). Caveolae are shear stress-sensitive signalling domains, and their distribution is not fixed: stimulation by shear stress redistributes them, with more caveolae reaching the cell surface under uniform shear than under static or disturbed conditions (55). Under uniform flow, and particularly during endothelial migration, caveolae concentrate at surface locations upstream of the flow direction, whereas under disturbed flow they show no positional preference (77). The endothelial cell, in other words, does not merely possess caveolae; it places them, and the placement is lost precisely where disease develops.

The functional consequence follows directly. Under disturbed flow, caveolin-1 becomes phosphorylated and binds eNOS, deactivating the enzyme, so the same haemodynamic input that scrambles caveolar positioning also suppresses nitric oxide output (78, 79). Direct evidence supports a role for caveolin-1 and caveolae in mechanotransduction and remodelling of blood vessels, which situates the caveola alongside the glycocalyx, junctional complexes, and Piezo1 as a genuine mechanosensor rather than a passive structure carried along by other sensors (80).

### 5.2 The glycocalyx as an upstream requirement

A finding that reframes the flow literature is that caveolar mechanosensitivity is conditional on the glycocalyx (54). Disturbed flow reduces caveolin-1 expression and abolishes most of its subcellular localisation preferences relative to uniform flow, and it downregulates caveolin-1 mechanosignalling, indicated by reduced colocalisation with serine 1,177 phosphorylated eNOS. Disturbed flow also inhibits glycocalyx expression. The decisive experiment is the enzymatic one. Heparan sulphate is a major glycocalyx component. In its absence, endothelial cells conditioned by uniform flow display near disturbed-flow-like caveolin-1 expression, localisation, and colocalisation with the active form of eNOS (54).

That result establishes an ordering. The glycocalyx is not a parallel sensor but an upstream requirement, and caveolar mechanosignalling is downstream of it. Uniform flow cannot protect a vessel whose glycocalyx has been stripped, which suggests that glycocalyx-based approaches targeted to regions of lesion development could prevent initiation and progression. It also offers a mechanistic account of why conditions that degrade the glycocalyx systemically, including hyperglycaemia and inflammatory states, accelerate atherosclerosis in vessel segments whose flow is otherwise favourable. The hierarchy running from flow pattern through glycocalyx integrity to caveolar positioning, eNOS coupling, and transport capacity is illustrated in Figure 4.

**Figure 4:**
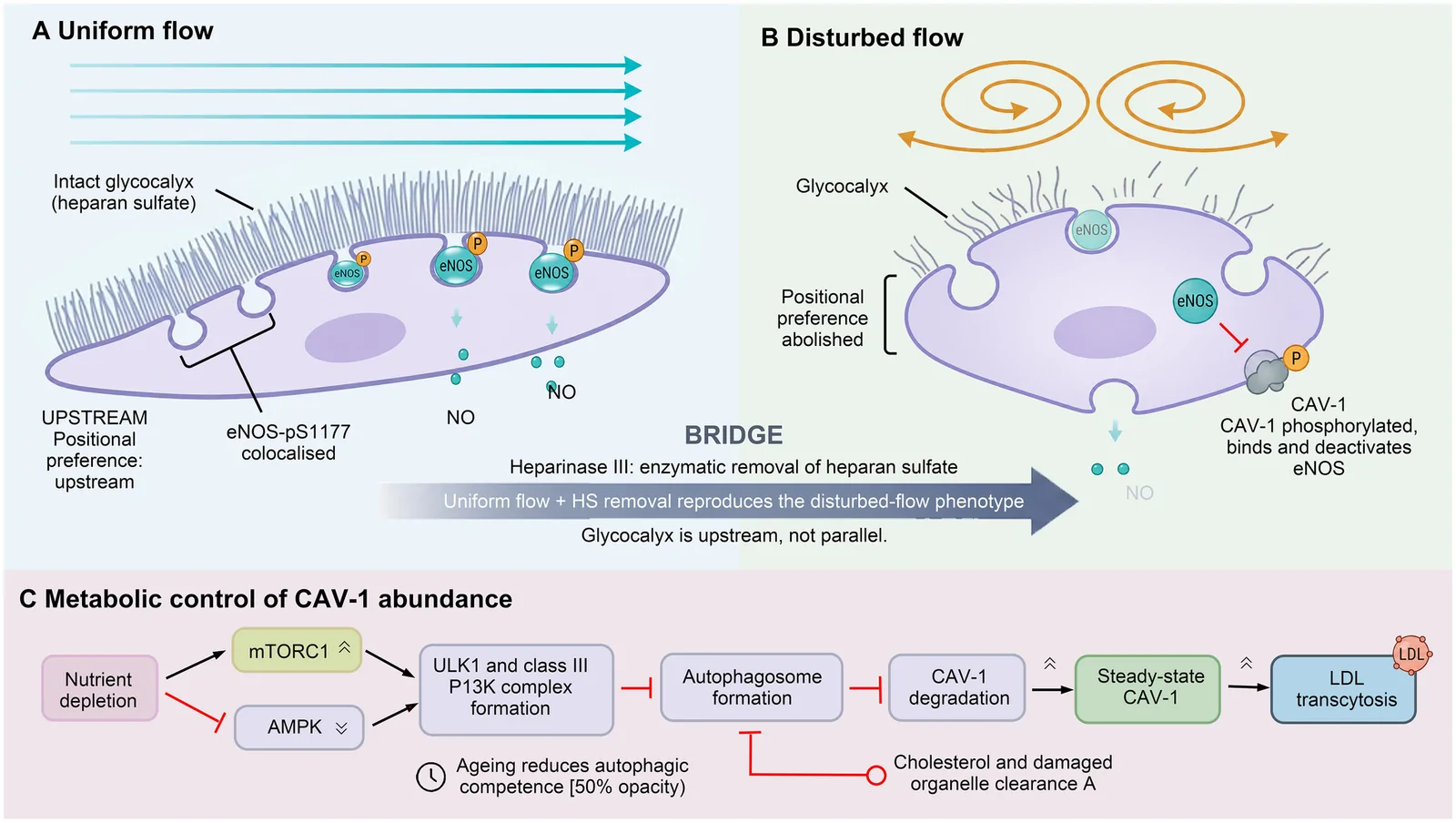
Hierarchical control of caveolar function by flow pattern, glycocalyx integrity, and autophagic turnover. Upstream regulation of caveolin-1 positioning and abundance. Under uniform flow, an intact glycocalyx permits caveolae to concentrate at surface locations upstream of the flow direction and to colocalise with serine 1,177 phosphorylated endothelial nitric oxide synthase, sustaining nitric oxide output. Under disturbed flow, glycocalyx expression falls, caveolin-1 expression declines, positional preference is abolished, and colocalisation with the active enzyme is reduced. Enzymatic removal of heparan sulphate reproduces the disturbed-flow phenotype in cells conditioned by uniform flow, establishing the glycocalyx as an upstream requirement rather than a parallel sensor. In parallel, nutrient depletion activates mTORC1 and inhibits AMPK, preventing formation of the ULK1 and class III PI3K complexes and reducing autophagic degradation of caveolin-1, which raises steady-state abundance and increases transcytosis.

### 5.3 Autophagy, nutrient sensing, and the abundance of Cav-1

Positioning is one control variable; abundance is the other, and abundance is set substantially by degradation. When endothelial cells sense nutrient depletion, they activate mTORC1 signalling and inhibit AMPK signalling, which prevents formation of the ULK1 and class III PI3K complexes and therefore of autophagic vesicles (53). The consequence is twofold: less protective autophagy to clear excess cholesterol and damaged organelles, and less degradation of Cav-1 through that route, and the reduced turnover of Cav-1 leads to increased LDL transcytosis across the endothelium. Autophagic flux, in short, sets steady-state Cav-1 and thereby sets transport capacity.

Autophagic competence declines with age (81), and its impairment raises cardiovascular risk, which supplies one plausible mechanism by which ageing raises lipoprotein entry without any change in plasma lipids. Related work in the intimal compartment is consistent: chaperone-mediated autophagy becomes impaired as disease progresses, which increases NLRP3 inflammasome activation and interleukin-1 beta secretion and thereby promotes vascular inflammation (82). Reduced Cav-1 clearance and heightened inflammasome output emerge from the same upstream defect, which is one of several places where the transport and immune arms of this review turn out to share a controller rather than merely to interact.

The shear stress and autophagy arms also intersect. Glycocalyx reduction under disturbed flow decreases caveolae and impairs colocalisation of caveolae with the active phosphorylated form of eNOS, attenuating nitric oxide signalling, while nutrient and mechanical inputs converge on the AMPK and mTORC1 axis that governs Cav-1 turnover (53, 54). A single endothelial cell at a bifurcation therefore experiences a coordinated shift: positional information lost, degradative capacity reduced, Cav-1 accumulating, nitric oxide falling, and transport rising. These are usually presented as four findings. They are better understood as one state.

## 6 Beyond the endothelium: immunometabolic remodelling of the vessel wall

### 6.1 Cav-1 in macrophage innate immune signalling

Once the particle is across, the endothelium ceases to be the only cell that matters. Multiple Toll-like receptors interact with plasma membrane components including Cav-1 to regulate phagocytosis and cell activation, which places Cav-1 in the innate immune circuit as well as the transport circuit (59). The direction of that effect is opposite to its endothelial one. Loss of Cav-1 has been associated with enhanced Toll-like receptor 4 and nuclear factor kappa B activity (47), and since TLR4 and NF-kappa B signalling enhances NLRP3 transcription (83–85) and thereby provides the priming signal, macrophage Cav-1 loss augments inflammasome priming under inflammatory conditions (40). Enhanced TLR4 and NF-kappa B activity has been linked to pro-inflammatory macrophage polarisation and NLRP3 priming. Interleukin-1 beta production rises and plaque destabilisation follows. The Cav-1 to TLR4 to NLRP3 axis has accordingly been proposed as a regulatory framework worth therapeutic exploration.

This sign reversal between compartments is, we think, the single most important structural fact about Cav-1 biology, and it is the reason that global manipulation has been unproductive. A systemic agent that lowered Cav-1 would reduce endothelial lipoprotein entry. The same agent would release a macrophage brake on inflammasome priming. The net effect would depend on the balance of these two opposing vectors in a given vessel at a given stage. That is not a therapeutic hypothesis so much as a coin toss. Compartment selectivity is therefore not a desirable feature of a future Cav-1 drug; it is the entire problem. Section 7.5 sets out the delivery approaches through which that requirement could be met in practice.

### 6.2 The inflammasome and the maturation of the lesion

The NLRP3 inflammasome comprises the NLRP3 receptor, the apoptosis-associated speck-like protein containing a caspase recruitment domain, and pro-caspase-1, and it assembles in monocytes, macrophages, and lymphocytes (61). Activation follows the familiar two-signal logic. Priming, signal one, proceeds through TLR phosphorylation and NF-kappa B translocation, which promotes NLRP3 transcription and expression of pro-interleukin-1 beta and pro-interleukin-18 (86). Triggering, signal two, promotes oligomerisation of NLRP3, the adaptor, and pro-caspase-1, and additional stimuli including reactive oxygen species promote assembly and caspase-1 activation, leading to processing and release of mature interleukin-1 beta (87, 88). Caspase-1 also enhances activation and release of gasdermin D, which executes pyroptosis.

The relevance to lipoprotein handling is direct. Oxidised LDL increases lactate dehydrogenase release and upregulates cleaved caspase-1, cleaved interleukin-1 beta, and gasdermin D in macrophages in an NLRP3-dependent manner, which closes the loop from transport to pyroptotic cell death and necrotic core formation (60). Interleukin-1 beta is an important proatherogenic factor, and the clinical significance of this axis is not hypothetical, since targeting interleukin-1 beta with a neutralising antibody proved beneficial for cardiovascular disease in the Canakinumab Anti-Inflammatory Thrombosis Outcomes Study. The inflammasome arm of the Cav-1 network is therefore the arm with the strongest existing human validation, though that validation was obtained without reference to Cav-1. The compartment-specific and directionally opposed actions of caveolin-1 in endothelium and macrophages, converging on NLRP3-dependent interleukin-1 beta output, are summarised in Figure 5.

**Figure 5:**
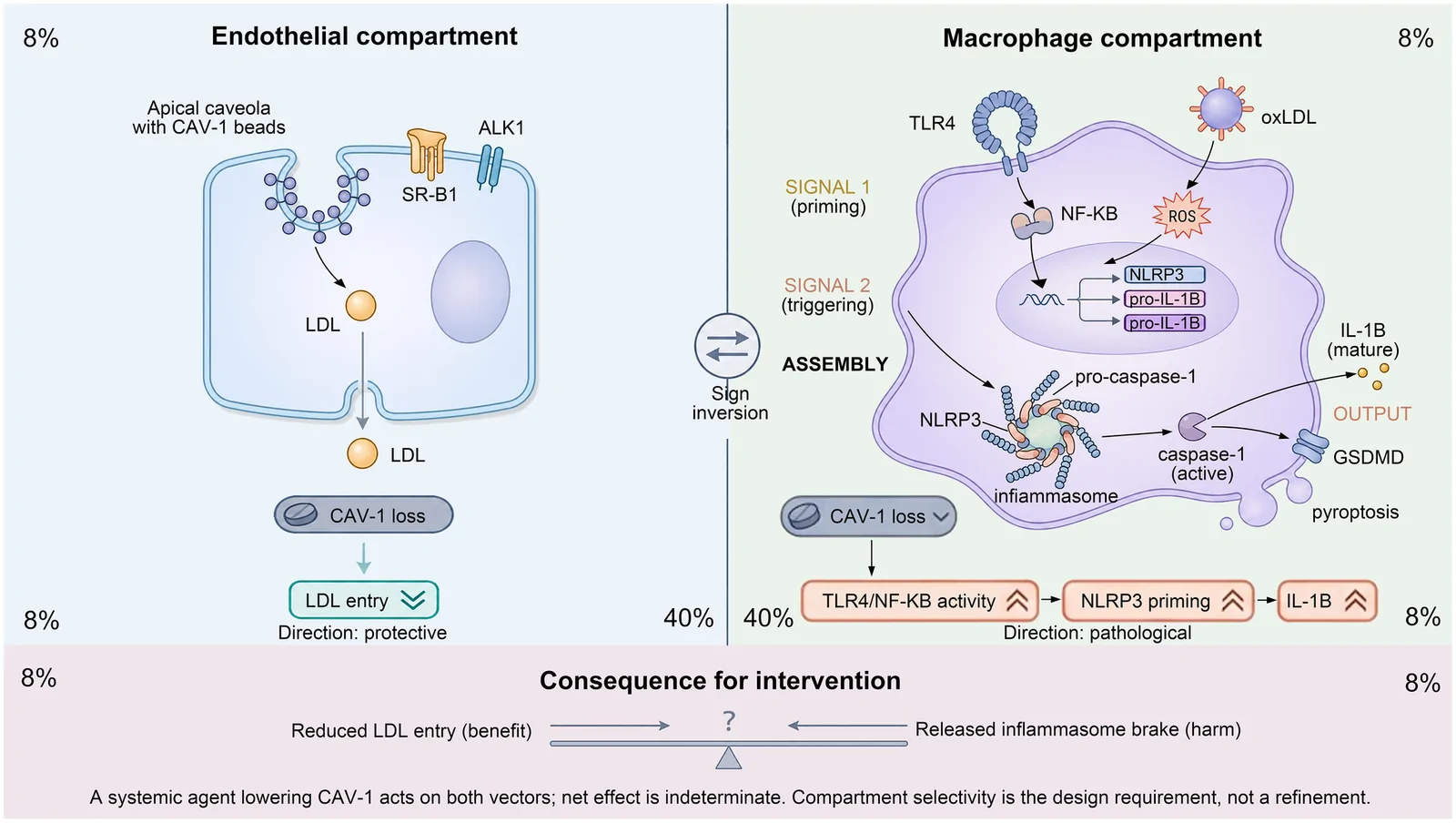
Opposing compartment-specific actions of caveolin-1 in endothelium and macrophages. The sign inversion that constrains therapeutic strategy. In endothelium, caveolin-1 promotes disease by enabling caveolae-dependent lipoprotein transcytosis, so that loss of caveolin-1 reduces lipoprotein entry. In macrophages, loss of caveolin-1 has been associated with enhanced Toll-like receptor 4 and nuclear factor kappa B activity, which augments transcriptional priming of NLRP3 and raises interleukin-1 beta output, so that loss of caveolin-1 acts in the opposite direction. The two-signal logic of the inflammasome is shown, with priming through Toll-like receptor 4 and nuclear factor kappa B driving NLRP3, pro-interleukin-1 beta, and pro-interleukin-18 expression, and triggering by oxidised lipoprotein and reactive oxygen species driving oligomerisation with the adaptor and pro-caspase-1, caspase-1 activation, interleukin-1 beta maturation, and gasdermin D-dependent pyroptosis. The lower bar illustrates why a systemic agent lowering caveolin-1 produces two opposing vectors of indeterminate net effect, and identifies compartment selectivity as the central design requirement.

### 6.3 Cholesterol handling, autophagy, and foam cell fate

Macrophage fate turns on the balance between cholesterol arriving and cholesterol leaving, and autophagy sits on both sides of that ledger. A moderate level of autophagy is beneficial during disease progression because it regulates inflammation and mediates macrophage cholesterol transport, whereas deficiency of autophagy proteins including ATG16L1 and ATG5 associates with increased interleukin-1 beta and platelet endothelial cell adhesion molecule 1, abnormal structures at endothelial contact sites, and increased neutrophil penetration leading to endothelial dysfunction (89). Conversely, ATG14 overexpression promotes fusion of macrophage autophagic vesicles with lysosomes through increased binding to syntaxin 17, and this promotes lipid degradation, regulates regulatory T cell populations, reduces apoptosis, and slows disease (53).

Cav-1 enters this ledger through cholesterol trafficking. Its expression is strictly related to internalisation and transcytosis of lipoproteins, and receptors for LDL and oxidised LDL localise in caveolae, where interaction with Cav-1 favours uptake and transcytosis of both. The trafficking consequences differ by route: when LDL is internalised through the LDL receptor it is degraded and cholesterol and receptor are recycled to the plasma membrane, but when the LDL receptor interacts with proprotein convertase subtilisin/kexin type 9 the complex is internalised through Cav-1 and the receptor is degraded (51). Cav-1 thus participates in setting receptor abundance itself, which extends its influence from the transport step to the cellular capacity for clearance.

### 6.4 Smooth muscle, matrix, and plaque stability

The final layer concerns whether a lesion becomes dangerous. As the intimal lesion progresses, macrophages proliferate beneath the intima and become foam cells, while proliferating smooth muscle cells secrete collagen to form the fibrous cap that stabilises the plaque, and the same cells secrete matrix metalloproteinases that promote rupture and increase instability (53). Activation of the subintimal inflammatory cascade through TLR, NLRP3, and PCSK9 pathways, combined with excessive cholesterol accumulation, triggers foam cell and leukocyte apoptosis and necrosis and produces a necrotic core rich in debris and lipid.

Cav-1 contributes here as well, through extracellular matrix remodelling and fibronectin deposition, which were identified alongside lipid trafficking and vascular inflammation as mechanisms by which Cav-1 controls diet-induced disease. This matters for the stage-dependence problem raised in Section 4. If Cav-1 influences matrix and stability independently of its transport role, then the failure of endothelial deletion to slow established lesions does not exhaust its relevance to advanced disease; it shows only that the transport function has ceased to be rate-limiting. Whether the matrix function remains addressable at that stage is an open and testable question, and one that no published study has been designed to answer (71). Cell-type-specific actions of caveolin-1 across the vascular wall, with direction of effect and supporting evidence, are compiled in Table 3.

**Table 3:** Cell-type-specific and stage-specific actions of caveolin-1 across the vascular wall, with direction of effect on disease.

#	Compartment	Process	Action attributed to caveolin-1 or caveolae	Direction on disease	Stage	Authors
1	Endothelium	Caveolae assembly	Caveolae cannot form without Cav-1. No coat means no transport machinery.	Permissive	All	Stephanos Pavlides, et al. (66)
2	Endothelium	Native LDL transcytosis	Cav-1 converts receptor binding into transcellular transport.	Pro-atherogenic	Early	Yifei Zhang, et al. (50)
3	Endothelium	Receptor concentration	Cav-1 concentrates SR-B1, ALK1, and LDL within one pocket. Effective capture rises.	Pro-atherogenic	Early	Israel O Bolanle, et al. (26)
4	Endothelium	Oxidised LDL transport	Cav-1 carries oxidised particles as well as native ones.	Pro-atherogenic	Early to mid	Francesca Luchetti (90)
5	Endothelium	Lipoprotein entry *in vivo*	Cav-1 deletion reduces LDL infiltration *in vivo*. An endothelial transgene reverses it.	Pro-atherogenic	Early	Sara Jeevanjee, et al. (91)
6	Endothelium	Progression of established lesions	Endothelial deletion does not inhibit progression once lesions are established.	Not rate-limiting	Late	Rocío Muñiz-Anquela, et al. (43)
7	Endothelium	eNOS scaffolding	Cav-1 holds eNOS in a low-activity state. Disturbed flow drives phosphorylation and deepens the inhibition.	Pro-atherogenic	All	Mohammad Hamrangsekachaee, et al. (55)
8	Endothelium	Nitric oxide dependence of protection	Protection survives loss of eNOS. Nitric oxide is not the mechanism.	Reinterprets mechanism	Early	Jonathan A C Sterne, et al. (92)
9	Endothelium	Mechanosensing and positioning	Caveolae concentrate upstream of the flow direction under uniform flow. Disturbed flow abolishes the preference.	Context-dependent	Pre-lesional	Mohammad Hamrangsekachaee, et al. (55)
10	Endothelium	Glycocalyx dependence	Cav-1 expression, localisation, and eNOS colocalisation require intact heparan sulfate.	Downstream of glycocalyx	Pre-lesional	Ian C Harding, et al. (54)
11	Endothelium	Autophagic turnover	Reduced autophagy lowers Cav-1 degradation. Steady-state Cav-1 and transcytosis both rise.	Pro-atherogenic	All	Yan Shu, et al. (53)
12	Endothelium	Src-dependent scission	Src phosphorylation of tyrosine 14 licences vesicle scission. The relay is demonstrated for Gp60-albumin.	Permissive	All	Israel O Bolanle, et al. (26)
13	Endothelium	PCSK9-dependent LDLR degradation	Cav-1 routes the LDL-LDLR-PCSK9 complex to receptor degradation.	Pro-atherogenic	All	Alessandra Puddu, et al. (51)
14	Endothelium	Extracellular matrix remodelling	Cav-1 influences matrix remodelling and fibronectin deposition.	Pro-atherogenic	Mid to late	Jonathan A C Sterne, et al. (92)
15	Endothelium	Adhesion molecule expression	Cav-1 deletion lowers adhesion molecule expression. Leukocyte capture falls.	Pro-atherogenic	Early to mid	Sara Jeevanjee, et al. (91)
16	Endothelium (cerebral)	Disposal setting	Brain endothelium degrades LDL through the same receptors that transport it elsewhere.	Atheroprotective	All	Sofia Kakava, et al. (27)
17	Endothelium (cerebral)	Suppression of caveolar transcytosis	Lipid transport-dependent suppression of caveolar transcytosis preserves barrier integrity.	Atheroprotective	All	Benjamin J Andreone, et al. (93)
18	Macrophage	Toll-like receptor regulation	Cav-1 restrains Toll-like receptor signalling. Its deletion raises pro-inflammatory output.	Atheroprotective	Mid to late	Ayyanar Sivanantham, et al. (59)
19	Macrophage	NLRP3 priming	Cav-1 loss enhances TLR4 and NF-kB activity. Inflammasome priming increases.	Atheroprotective	Mid to late	Li Qin, et al. (52)
20	Macrophage	Polarisation and plaque stability	Enhanced TLR4 and NF-kB activity promotes pro-inflammatory polarisation. IL-1B rises and plaques destabilise.	Atheroprotective	Late	Xuan Liu, et al. (40)
21	Macrophage	Foam cell formation	Caveolar receptors participate in oxidised LDL uptake. Lipid loading drives foam cell formation.	Pro-atherogenic	Mid	Yifei Zhang, et al. (50)
22	Macrophage	Pyroptotic death and necrotic core	Oxidised LDL activates caspase-1, IL-1B, and gasdermin D through NLRP3.	Pro-atherogenic	Late	Ruosen Yuan, et al. (60)
23	Macrophage	Cholesterol efflux and autophagy	Moderate autophagy sustains macrophage cholesterol export and restrains inflammation.	Atheroprotective	Mid to late	Ismail Sergin, et al. (94)
24	Smooth muscle	Fibrous cap formation	Proliferating smooth muscle secretes collagen. The fibrous cap stabilises the plaque.	Atheroprotective	Late	Grootaert MOJ, et al. (95)
25	Smooth muscle	Matrix metalloproteinase secretion	Smooth muscle metalloproteinases degrade the cap. Rupture risk rises.	Pro-atherogenic	Late	Yan Shu, et al. (53)
26	Vessel wall	Mechanotransduction and remodelling	Cav-1 and caveolae mediate mechanotransduction and vascular remodelling.	Context-dependent	All	Demitri F Papolos, et al. (96)
27	Systemic	Global deletion phenotype	The global knockout is systemically abnormal. Endothelial re-expression rescues it.	Confounded	All	Curry-Koski T, et al. (97)
28	Endothelium	Sex dependence	Oestrogen inhibits caveolar transcytosis. Early disease is lower in females.	Modifier	Early	Rocío Muñiz-Anquela, et al. (43)
29	Endothelium	Sex dependence of lipid transport phenotype	Endothelial Cnr1 deletion lowers CAV1 and ALK1. Effects are stronger in females.	Modifier	Early	Bingni Chen, et al. (57)
30	Endothelium	Nanocarrier uptake	Caveolar endocytosis enhances nanocarrier uptake. The pathogenic route becomes a delivery route.	Exploitable	All	Yifei Zhang, et al. (50)

Direction on disease denotes the effect of the caveolin-1-dependent process as reported, not the effect of deleting caveolin-1; readers comparing rows should note that a pro-atherogenic process implies that loss of caveolin-1 is protective in that compartment, and the reverse for an atheroprotective one. The opposing directions in endothelium and macrophage constitute the sign inversion discussed in Section 6.1 and are the principal reason global deletion models yield uninterpretable net phenotypes. Stage is assigned according to the lesion stage at which the study interrogated the process, and entries marked All indicate that stage-specific data are not available rather than that stage-independence has been demonstrated. Entries marked Confounded or Not rate-limiting record findings that constrain interpretation rather than establish a direction. Abbreviations are as defined in Table 1.

## 7 A molecule-centred disease network

### 7.1 Assembling the circuit

The material reviewed so far is usually partitioned into four literatures: caveolar structure (98), lipoprotein transcytosis (99), endothelial mechanobiology (55), and macrophage immunometabolism (40). Read as a network they describe a single control system with Cav-1 at its centre, and the system has a recognisable shape.

The input layer is physical and metabolic. Flow pattern determines glycocalyx integrity, glycocalyx integrity permits caveolar positioning and coupling to eNOS, and nutrient status acting through AMPK and mTORC1 determines autophagic flux and therefore Cav-1 abundance (100). The processing layer is the caveola itself, where SR-B1 and ALK1 capture lipoprotein, DOCK4 and RAC1 commit the vesicle, and Src-dependent phosphorylation of Cav-1 licences scission (26). The output layer is the intima, where retained and oxidised particles drive chemokine production, monocyte recruitment, TLR4 and NF-kappa B priming, NLRP3 assembly, interleukin-1 beta release, pyroptosis, and eventually matrix remodelling that determines whether the plaque holds.

Two features of this network are worth stating because they are not apparent from any single arm. First, it contains a positive feedback loop of real gain. Reduced nitric oxide and heightened inflammation degrade the glycocalyx and impair autophagy, which raises Cav-1 and raises transport, which delivers more substrate and more inflammation (101). A system with this topology does not need a large initiating perturbation to reach a pathological steady state, which may explain why lesion location is so reproducible across individuals with widely different plasma lipids. Second, the network contains a sign inversion at the macrophage node, where Cav-1 restrains rather than promotes pathology (41). A network with a hub whose sign flips between compartments will resist any intervention applied to the hub globally, and that is precisely what the therapeutic history shows. The integrated caveolin-1 network, comprising mechanical and metabolic inputs, caveolar processing, and immunometabolic output with the feedback loop that stabilises the diseased state, is presented in Figure 6.

**Figure 6:**
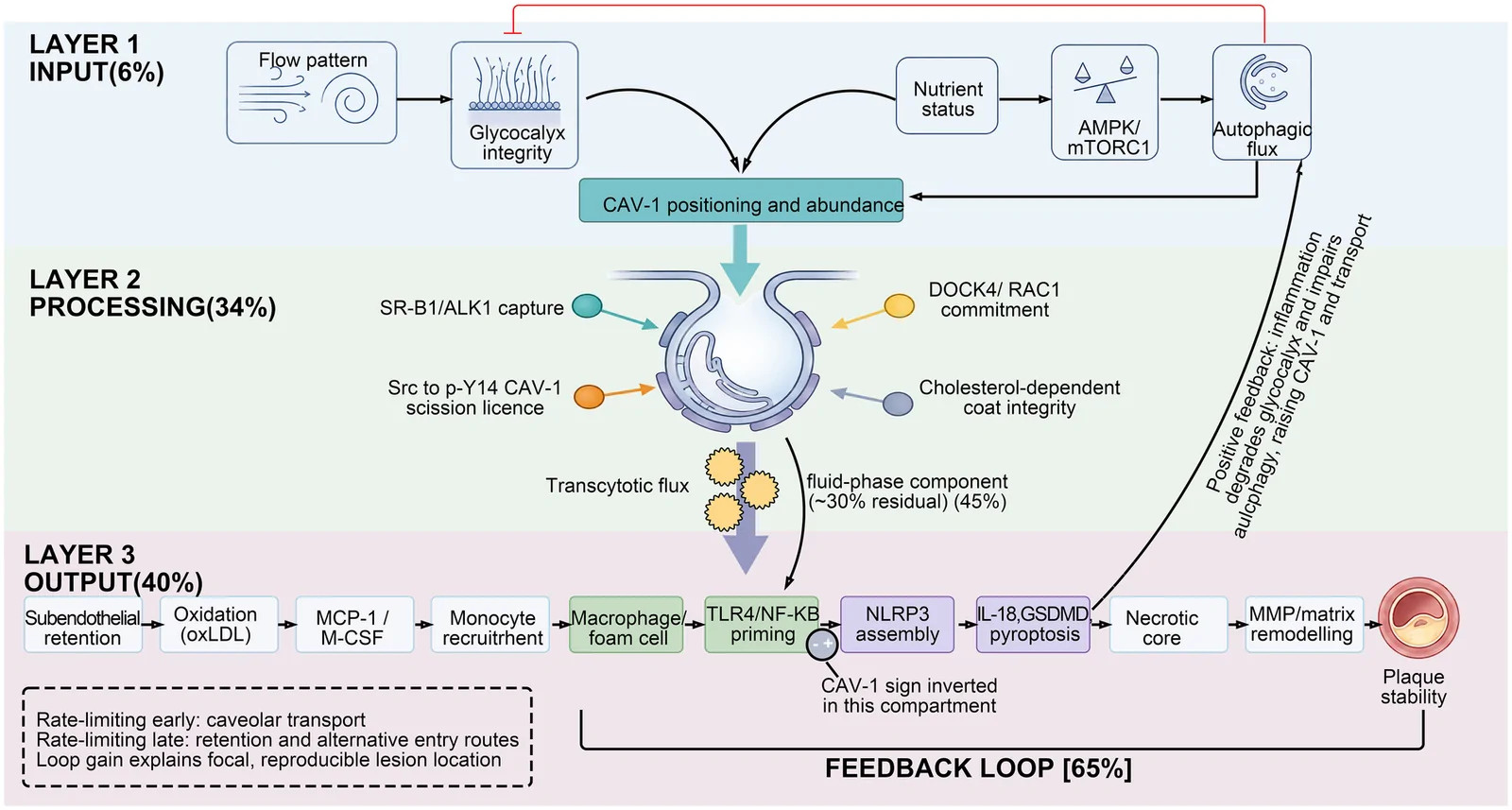
The integrated caveolin-1 network linking biomechanical and metabolic input to immunometabolic output. A molecule-centred disease network assembled from the evidence reviewed. The input layer comprises flow pattern acting through glycocalyx integrity and nutrient status acting through the AMPK and mTORC1 axis on autophagic flux, both converging on caveolin-1 positioning and abundance. The processing layer is the caveola, where scavenger receptor class B type 1 and activin receptor-like kinase 1 capture lipoprotein, the dedicator of cytokinesis 4 and RAC1 module commits the vesicle, and Src-dependent phosphorylation licences scission. The output layer is the intima, comprising retention, oxidation, monocyte recruitment, inflammasome priming and activation, interleukin-1 beta release, and matrix remodelling that determines plaque stability. A feedback loop, shown in amber, returns from inflammatory output to glycocalyx degradation and impaired autophagy, raising caveolin-1 and transport further, which supplies the network with positive gain and accounts for the reproducibility of lesion location. The macrophage node carries an inverted sign, marked accordingly.

### 7.2 What the network explains that single pathways do not

The value of the network framing is that it accounts for observations that are anomalous within any single arm. Focal lesion distribution despite systemic lipid exposure follows from the mechanical input layer rather than from lipoprotein concentration. The dissociation of atheroprotection from nitric oxide production, established by the triple knockout, follows from the transport arm operating in parallel with the eNOS arm rather than through it. The stage-dependence of endothelial Cav-1 deletion follows from the observation that once the barrier is compromised and neovascularisation is established, the caveolar route loses its monopoly and ceases to be rate-limiting (43). The opposing macrophage phenotype follows from the sign inversion. None of these requires an auxiliary hypothesis once the network is drawn.

### 7.3 Therapeutic tractability: three routes and their honest odds

The first route is transport-selective interference. Its logic is strong, since the SR-B1 trafficking function is structurally separable from its ligand-binding function, and blocking the eight-residue cytoplasmic motif or the DOCK4 interaction would suppress entry while sparing hepatic reverse cholesterol transport. Its obstacles are equally real: protein-protein interaction interfaces of this kind are difficult to drug, endothelial selectivity would have to be engineered rather than assumed, and the stage-dependence data suggest the benefit would be confined to primary prevention. This is the most mechanistically defensible route and the least commercially straightforward one.

The second route is upstream restoration rather than downstream blockade. Given that glycocalyx integrity is required for caveolar mechanosignalling, a glycocalyx-based therapeutic targeted to areas of disease development could prevent initiation and progression, and given that autophagic flux sets Cav-1 abundance, agents that restore flux would lower transport without touching Cav-1 directly. Salidroside, which suppresses transcytosis through autophagic targeting of active Src and Cav-1, is the existing proof of principle. The appeal is that these approaches work with endogenous regulation rather than against it; the difficulty is that both glycocalyx repair and autophagy induction are pleiotropic, and neither has a validated vascular readout that would support a trial.

The third route inverts the problem and is, paradoxically, the nearest to application. Rather than suppressing caveolar uptake, it exploits it. Nanocarriers bearing small interfering RNA or DNA can enhance transfection efficiency through caveolin-mediated endocytosis, and a concrete implementation has been reported in which vascular cell adhesion molecule 1 binding peptide-targeted cationic liposomes carrying methylated NLRP3 siRNA selectively delivered their cargo to inflammatory endothelial cells, inhibiting NLRP3 expression *in vitro* and *in vivo* and thereby blocking tumour necrosis factor alpha-induced inflammasome activation and Cav-1-mediated LDL transcytosis. Two features make this attractive. First, the delivery route is the disease route, so targeting and pathology coincide. Second, adhesion molecule expression restricts delivery to activated endothelium, which supplies the compartment selectivity that Section 6.1 identified as the central requirement. The application of Cav-1 in nanomedicine accordingly offers a genuinely novel perspective on treatment, though the usual caveats regarding nanoparticle biodistribution, immunogenicity, and manufacturing apply with full force. Therapeutic strategies that target caveolin-1 or caveolae-dependent transport, with mechanism, developmental stage, and principal limitation, are summarised in Table 4.

**Table 4:** Strategies targeting caveolin-1 or caveolae-dependent lipoprotein transport, with mechanism, developmental status, and principal limitation.

#	Strategy or agent	Class	Mechanism	Status	Principal limitation	Authors
1	Filipin	Sterol-binding agent	Sequesters cholesterol and disassembles caveolae; reduces oxidised LDL transport by 72.4%	Probe only	Non-selective; disrupts all cholesterol-dependent membrane domains; not usable *in vivo*	Maddila SC, et al. (102)
2	Carrageenan	Coat disruptor	Interferes with caveolar coat; reduces oxidised LDL transport by 48.9%	Probe only	Non-selective and pro-inflammatory in its own right	Shao-wei Sun, et al. (52)
3	Nocodazole	Microtubule agent	Blocks microtubule-dependent vesicular transit; reduces oxidised LDL transport by 79.8%	Probe only	Blocks all microtubule-dependent traffic; cytotoxic	Sun SW, et al. (52)
4	Salidroside	Natural product	Autophagic targeting of active Src and Cav-1 suppresses LDL transcytosis	Preclinical	Selectivity and effect size *in vivo* untested; mechanism is indirect	Xiangli Bai, et al. (103)
5	SR-B1 to DOCK4 interface inhibition	Protein interaction inhibitor	Occludes the 8-amino-acid cytoplasmic motif recruiting DOCK4, abolishing trafficking while sparing ligand binding	Conceptual	Protein interaction interfaces are difficult to drug; endothelial selectivity must be engineered	Linzhang Huang, et al. (31)
6	SR-B1 ligand blockade	Receptor antagonist	Prevents LDL binding to SR-B1, reducing capture	Not viable	Compromises hepatic reverse cholesterol transport; the beneficial and pathogenic functions share the binding site	Liming Yu, et al. (104)
7	Combined SR-B1 and ALK1 inhibition	Dual receptor blockade	Reduces *in vivo* LDL transport by approximately 70%	Conceptual	Residual fluid-phase flux persists; ALK1 has essential roles in vascular development	Israel O Bolanle, et al. (26)
8	Global Cav-1 suppression	Target knockdown	Removes the caveolar platform, lowering transport	Not viable	Sign inversion: releases the macrophage inflammasome brake; net effect indeterminate	Xuan Liu, et al. (40)
9	Endothelial-restricted Cav-1 suppression	Targeted knockdown	Lowers transport in the causal compartment while sparing macrophage Cav-1	Conceptual	Ineffective once lesions are established; confined to primary prevention	Rocío Muñiz-Anquela, et al. (43)
10	Glycocalyx repair	Upstream restoration	Restores heparan sulfate, permitting caveolar positioning and eNOS coupling under favourable flow	Conceptual	Pleiotropic; no validated vascular readout to support a trial	Israel O Bolanle, et al. (26)
11	Autophagy induction	Upstream restoration	Raises autophagic flux, increasing Cav-1 degradation and lowering transport	Conceptual	Pleiotropic; therapeutic window uncertain since excessive autophagy is also harmful	Xinbo Zhang, et al. (105)
12	AMPK activation	Metabolic modulator	Opposes mTORC1, restoring ULK1 and class III PI3K complex formation and Cav-1 turnover	Conceptual	Systemic metabolic effects; endothelial specificity absent	Claudia Rodríguez, et al. (106)
13	Src kinase inhibition	Kinase inhibitor	Prevents tyrosine 14 phosphorylation of Cav-1, withholding the scission licence	Conceptual	Src is broadly required; oncology-grade toxicity unacceptable for prevention	Israel O Bolanle, et al. (26)
14	NLRP3 siRNA in VCAM-1-targeted liposomes	Targeted nanomedicine	Delivers methylated NLRP3 siRNA selectively to inflamed endothelium, blocking inflammasome activation and Cav-1-mediated transcytosis	Preclinical, *in vivo*	Nanoparticle biodistribution, immunogenicity, and manufacturing; durability unknown	Xiong Jia, et al. (107)
15	Caveolin-mediated nanocarrier delivery	Delivery platform	Exploits caveolar endocytosis to raise transfection efficiency, using the disease route as the delivery route	Preclinical	Depends on the very pathway being targeted; competition with lipoprotein cargo untested	Yifei Zhang, et al. (50)
16	PCSK9 inhibition	Approved lipid therapy	Prevents PCSK9-driven, Cav-1-dependent degradation of the LDL receptor. The degradative LDLR route is preserved, shifting particle fate from preservation toward consumption.	Approved	Endothelial action at clinically achievable concentrations is untested; the established site of action is hepatic	Alessandra Puddu, et al. (51)
17	IL-1B neutralisation	Approved biologic principle	Neutralises the terminal cytokine of the inflammasome arm of the network	Clinically validated	Acts downstream of transport; infection risk; does not address entry	Yan Shu, et al. (53)
18	NLRP3 inflammasome inhibition	Small molecule	Blocks assembly, lowering IL-1B and gasdermin D-dependent pyroptosis	Preclinical to clinical	Downstream of entry; compartment selectivity between endothelium and macrophage unresolved	Na Lu, et al. (61)
19	Oestrogen-mediated transcytosis inhibition	Endogenous modifier	Inhibits caveolar transcytosis, plausibly contributing to lower early disease in females	Observational	Systemic hormonal exposure unacceptable as a preventive strategy	Rocío Muñiz-Anquela, et al. (43)
20	Endothelial CB1 antagonism	Receptor antagonist	Lowers CAV1 and ALK1 transcription and reduces apical caveolae, reducing lipid transport	Preclinical	Central cannabinoid receptor blockade carries established neuropsychiatric liability	Bingni Chen, et al. (57)
21	Piezo1 modulation	Mechanosensor modulator	Interrupts disturbed-flow-induced endothelial inflammation upstream of caveolar dysfunction	Preclinical	Piezo1 is broadly expressed; clinical efficacy and safety not yet evaluated	Yining Lan, et al. (67)
22	Transport-to-degradation redirection	Hypothesis	Imposes the cerebral endothelial disposal setting on arterial endothelium, routing LDL to lysosome rather than transcytosis	Hypothesis	Specifying factor unidentified; requires that uptake and fate be measured separately	Sofia Kakava, et al. (27)
23	Exocytic step blockade	Hypothesis	Blocks the terminal fusion event, the most downstream point at which entry could be prevented	Hypothesis	Fusion machinery entirely uncharacterised; no candidate target exists	Yifei Zhang, et al. (50)
24	Statin therapy	Approved lipid therapy	Lowers circulating LDL and, through depletion of membrane free cholesterol, lowers endothelial Cav-1 abundance and restores eNOS function	Approved	Transcytosis has never been measured *in vivo* under statin therapy; the Cav-1 effect is secondary to cholesterol synthesis inhibition	Israel O Bolanle, et al. (26); Olivier Feron, et al. (108, 109); Michel Pelat, et al. (110)
25	SGLT2 inhibition	Approved glucose-lowering therapy	Limits pathological endothelial caveolin-1 and PV-1 induction in diabetic glomerular endothelium; activates AMPK and restrains mTORC1, the axis that sets autophagic Cav-1 turnover	Approved for other indications; unstudied in artery	Direct evidence is renal rather than arterial; no measurement of arterial Cav-1 or LDL transcytosis exists	Marco Locatelli, et al. (111)

Status denotes the furthest stage of development reported for the strategy as applied to caveolin-1-dependent lipoprotein transport specifically, not the regulatory status of the agent for other indications; statins, PCSK9 inhibitors, and interleukin-1 beta neutralisation are approved or clinically validated for cardiovascular disease but none was developed against the transport step. Entries marked Conceptual are mechanistically supported but have no reported agent, and entries marked Hypothesis are advanced in this review and have not been tested. Entries marked Not viable are included because their limitations are instructive rather than because they remain under consideration. Percentage values are reported as stated in the source. Abbreviations are as defined in Table 1.

### 7.4 Do established cardiovascular drugs act on the caveolin-1 axis?

No approved cardiovascular drug was designed against caveolar transport. Three classes nonetheless intersect the pathway, and the evidence for each sits at a different level. Setting them out separately is worthwhile, because a repurposing hypothesis built on an approved agent is a far cheaper experiment than a novel entity, and because two of the three generate predictions that could be tested in existing cohorts.

Statins have the most direct link, and it follows from the cholesterol dependence established in Section 2.3. Caveolin transcription and protein abundance track membrane free cholesterol. Hypercholesterolaemia raises Cav-1 and its association with eNOS, and nitric oxide production falls as a result (108). Inhibition of HMG-CoA reductase does the reverse: caveolin abundance falls in proportion to the reduction in intracellular free cholesterol, and eNOS function improves both *in vitro* and *in vivo* (109). Rosuvastatin lowers Cav-1 and improves nitric oxide-dependent heart rate and blood pressure variability in apolipoprotein E-deficient mice (110), and the eNOS-dependent vascular effects of atorvastatin are likewise accompanied by decreased Cav-1. The implication for this review is specific rather than general. A statin lowers the substrate, which is plasma LDL, and it plausibly also lowers the endothelial transport capacity that handles the substrate. If the second action contributes materially to outcome, then part of the benefit of statin therapy is already transport-directed, and the mechanism reviewed here would have its first clinical footing. Two caveats prevent us from asserting this. Endothelial LDL transcytosis has never been measured *in vivo* under statin therapy, and the effect on Cav-1 is a downstream consequence of cholesterol synthesis inhibition rather than a designed action on the caveola.

PCSK9 inhibitors intersect the pathway at the level of particle fate rather than particle entry. When the LDL receptor is engaged by PCSK9, the complex is internalised through a Cav-1-dependent route that ends in receptor degradation, whereas the receptor alone recycles and the particle is consumed in the lysosome (52). Blocking PCSK9 therefore preserves the degradative route and the receptor pool that serves it. In the terms of the hypothesis advanced in Section 8.3, PCSK9 inhibition shifts the endothelial disposal setting from preservation toward consumption, which is the direction this review argues is therapeutically desirable. Whether this occurs in arterial endothelium at clinically achievable concentrations is untested, since the established site of action of these agents is hepatic. The experiment that would answer it is straightforward: measure uptake and fate separately in arterial endothelial cells under PCSK9 blockade, rather than measuring plasma LDL.

SGLT2 inhibitors have the weakest direct evidence and the most interesting indirect case. The direct data are renal. In diabetic mice the glomerular endothelium showed increased caveolin-1 and plasmalemma vesicle-associated protein together with loss of fenestration, and empagliflozin limited these abnormalities through reduced podocyte-derived VEGF-A signalling (111). That is a different vascular bed and a different disease process, and it cannot be transferred to the artery without evidence. The indirect case rests on the AMPK and mTORC1 axis. Section 5.3 established that this axis governs autophagic degradation of Cav-1 and thereby sets transport capacity, and SGLT2 inhibitors activate AMPK and restrain mTORC1 in vascular and cardiac tissue. Hyperglycaemia and advanced glycation end product signalling raise endothelial Cav-1 and accelerate transcytosis, so a substrate for benefit plausibly exists in diabetes. All of this remains inference. No study has measured arterial Cav-1 abundance or LDL transcytosis under SGLT2 inhibition, and we flag the gap as a concrete and readily addressable experiment rather than as a conclusion.

Two further agents deserve brief comment. Metformin and other AMPK activators touch the same turnover node as the salidroside data discussed in Section 4.5, which makes them mechanistically continuous with the upstream restoration route rather than a separate proposal. Ezetimibe is sometimes cited in this context because an annexin A2-caveolin-1 complex was once proposed as its target; that report has been retracted, Niemann-Pick C1-like 1 is the accepted target (112), and we therefore do not treat ezetimibe as evidence for caveolar pharmacology. Table 4 has been extended to record the three classes above with their status and limitations.

### 7.5 Making compartment selectivity concrete: delivery strategies

Section 6.1 argued that compartment selectivity is not a refinement of a future Cav-1 drug but its central design problem, and a reader is entitled to ask what a solution would actually look like. The pharmacological answer is unpromising on its own. Cav-1 is an intracellular structural protein with no ligand-binding pocket, so selectivity cannot come from receptor pharmacology and must come from delivery. That reframing is useful, because delivery to activated endothelium and to plaque macrophages is an engineering problem with an established literature rather than an open biological question. Four families of approach are available, and they differ in how far they have been taken. Table 5 sets them out with their payloads and limitations.

**Table 5:** Delivery approaches capable of delivering compartment-selective modulation of the caveolin-1 axis, with candidate payloads and principal limitations.

#	Platform	Targeting moiety	Compartment addressed	Candidate payload for the Cav-1 axis	Status	Principal limitation
1	Cationic liposome	VCAM-1-binding peptide (VHPKQHRGGSKGC)	Activated endothelium	NLRP3 siRNA, demonstrated; *CAV1* siRNA, proposed	Preclinical, *in vivo*	Durability, repeat dosing, and biodistribution unresolved
2	Antibody-directed conjugate	Anti-VCAM-1 or anti-ICAM-1	Activated endothelium	siRNA, antisense oligonucleotide, small molecule	Preclinical	Antigen is not artery-restricted; any inflamed endothelium is addressed
3	Polymeric or lipid nanoparticle, multiplexed siRNA	Formulation-driven endothelial uptake	Vascular endothelium	Adhesion molecule silencing; *CAV1* knockdown	Preclinical, *in vivo*	Efficiency low relative to hepatic delivery
4	Ligand-directed conjugate	Receptor-specific ligand (ASGPR precedent)	Hepatocyte, as precedent only	siRNA	Approved for hepatic targets	No endothelial receptor of equivalent affinity and internalisation is known
5	Macrophage-directed nanoparticle	Mannose, dextran, S2P peptide, HDL mimetic	Plaque macrophage	Inflammasome inhibitor or autophagy inducer; never *CAV1* knockdown	Preclinical	Sign inversion makes payload selection decisive
6	Cell membrane-coated carrier	Neutrophil or platelet membrane	Activated endothelium	Antisense oligonucleotide, siRNA	Preclinical	Manufacturing reproducibility and batch consistency
7	Engineered extracellular vesicle	VCAM-1 peptide display	Activated endothelium	siRNA, small molecule	Preclinical	Loading efficiency and standardisation
8	Shear-responsive carrier	Abnormal wall shear stress	Lesion-prone geometry	Any of the above	Early preclinical	Trigger threshold and spatial specificity unvalidated in artery
9	Interface-directed agent	SR-B1 cytoplasmic motif or DOCK4 interface	Endothelium	Peptidomimetic or targeted degrader	Conceptual	Intracellular delivery to endothelium unsolved; interface is difficult to drug

Status refers to the furthest stage reported for the platform in atherosclerosis or in a directly comparable vascular application, not to its status for other indications. Candidate payloads for the Cav-1 axis are proposed by us except where a demonstrated payload is named. Entries 1 and 5 must carry opposite payloads because of the compartment sign inversion established in Section 6.1. ASGPR, asialoglycoprotein receptor; HDL, high-density lipoprotein; ICAM-1, intercellular adhesion molecule 1; other abbreviations are as defined in Table 1.

The first family is ligand-directed targeting of activated endothelium. Vascular cell adhesion molecule 1 is induced early and focally at lesion-prone sites, which makes it the natural address. The VCAM-1-binding peptide with sequence VHPKQHRGGSKGC has been used to direct cationic liposomes carrying methylated NLRP3 small interfering RNA to inflammatory endothelium *in vitro* and *in vivo*, inhibiting inflammasome activation and Cav-1-mediated LDL transcytosis (107), and the same peptide has been developed as a positron emission tomography ligand, which supplies a companion imaging readout that a first-in-human study would require. Antibody-directed conjugates against VCAM-1 or intercellular adhesion molecule 1 achieve comparable addressing, and multiplexed small interfering RNA against several endothelial adhesion molecules has reduced leukocyte recruitment and vascular inflammation in mice (113). For the pathway reviewed here the payload would change rather than the platform: small interfering RNA against CAV1, or an agent directed at the SR-B1 cytoplasmic motif, in place of an inflammasome target.

The second family is macrophage-directed delivery, and here the sign inversion dictates the design. Because macrophage Cav-1 restrains Toll-like receptor and inflammasome output, a carrier that reached plaque macrophages must not carry Cav-1 knockdown; it would worsen precisely what the endothelial arm is meant to prevent. The appropriate payloads for that compartment are inflammasome inhibitors, autophagy-restoring agents, or efflux promoters. Mannose- and dextran-functionalised particles, the S2P peptide, high-density lipoprotein mimetics, and macrophage-tropic lipid nanoparticles all reach plaque macrophages with reasonable efficiency (114). The practical conclusion is that a Cav-1 programme requires two carriers with opposite payloads rather than one, which is unusual but not prohibitive.

The third family concerns modality and chemistry rather than address. Conjugate chemistry has already produced a chronic cardiovascular nucleic acid therapy, since N-acetylgalactosamine-conjugated small interfering RNA achieves durable hepatocyte delivery through the asialoglycoprotein receptor with twice-yearly dosing. The lesson is encouraging and the obstacle is specific: no endothelial receptor with comparable expression, affinity, and internalisation rate has been identified, and until one is, ligand-directed conjugates for endothelium remain aspirational. Lipid nanoparticle tropism can instead be tuned by formulation, and adeno-associated virus capsids with endothelial tropism exist, but neither approach yet delivers arterial endothelial specificity at the level that hepatocyte targeting reaches routinely. We regard the identification of an endothelium-restricted internalising receptor as the principal unsolved engineering problem for this field.

The fourth family exploits the pathway rather than avoiding it. Caveolin-mediated endocytosis enhances nanocarrier transfection efficiency, so the route that delivers lipoprotein into the wall is also the route that delivers the carrier into the cell. That coincidence is attractive and carries an internal tension worth stating: a carrier that depends on caveolae for entry will lose potency in proportion to its own success in suppressing them. Designs that suppress the SR-B1 to DOCK4 interaction rather than Cav-1 itself avoid this trap, since they leave the vesicular machinery intact while removing the guided lipoprotein input. Physical and biological addressing supplies a further layer. Shear-responsive carriers activate where wall shear stress is abnormal, which coincides with the geometry that determines lesion location; cell membrane-coated carriers inherit the natural tropism of neutrophils or platelets for activated endothelium; and engineered extracellular vesicles displaying a VCAM-1 peptide combine biological tolerance with directed addressing.

The constraints deserve to be as concrete as the proposals. Most intravenously administered nanocarriers accumulate in liver and spleen, and arterial endothelial delivery efficiency remains low in absolute terms. VCAM-1 is expressed on activated endothelium wherever inflammation occurs, so compartment selectivity in this setting means selectivity for activated endothelium rather than for artery. Chronic administration raises immunogenicity, accumulation, and cost, and the stage-dependence established in Section 4.3 means the target population is asymptomatic people in primary prevention, which sets a safety threshold higher than any oncology nanomedicine has had to meet. Those four constraints, rather than any gap in mechanism, are why we described this route in Section 7.3 as the nearest to application while still being some distance from it.

### 7.6 What would change our minds

A review of this kind should state what evidence would falsify its central claim. If inducible endothelial deletion of Cav-1 in an established lesion model reduced matrix metalloproteinase activity and improved cap thickness without altering lipoprotein entry, the transport-centred framing would be incomplete and the matrix arm would need equal billing. If human carotid endarterectomy specimens showed no relationship between caveolar density and regional lipoprotein retention, the mouse data would have to be regarded as species-specific. If a selective DOCK4 interaction inhibitor suppressed transcytosis without affecting lesion area, the necessity of the transport step for disease would be in question. Each of these experiments is feasible with existing tools, and none has been reported.

## 8 Open questions and future directions

### 8.1 The four questions that matter most

The first is the exocytic step. LDL reaches the abluminal side and enters the subendothelial space through exocytosis, and the specific mechanism of this process requires further investigation. This is a substantial gap, since the fusion step is the last committed event before retention and would be the most downstream point at which entry could be blocked. Nothing is known about the fusion machinery involved, its regulation, or whether it is shared with other transcytotic cargo.

The second is the quantitative partition between receptor-guided and fluid-phase transport. Combined loss of SR-B1 and ALK1 reduces transport by roughly seventy per cent, leaving a residual that has never been mechanistically assigned (26). Since up to seven particles can be close-packed within a single caveola, and since ligand-triggered kinase relays such as the Gp60 and Src pathway may increase transport of co-engulfed solutes as well as of bound ligand, eliminating receptors may reduce fluid-phase transport in addition to receptor-mediated transport, which would account for some of the confusion in the field. Resolving this requires cargo-tracking approaches at single-vesicle resolution rather than more knockouts.

The third is vascular bed specificity, which is more instructive than it appears. Brain endothelial cells, unlike aortic endothelial cells, do not transport but instead degrade LDL through both the LDL receptor and ALK1, and blood-brain barrier permeability is regulated by lipid transport-dependent suppression of caveolae-mediated transcytosis. The same receptors, in a different endothelium, produce the opposite outcome. Whatever specifies this difference is a natural switch between transport and degradation, and a therapeutic that could impose the cerebral setting on the aorta would address the disease at its origin. Identifying that specifying factor strikes us as the highest-value open problem in the field.

The fourth is the human question. Essentially all causal evidence derives from mice, and the differences are not cosmetic: murine and human lipoprotein profiles differ, murine lesions rarely rupture, and the receptor contributions quantified *in vivo* were established at least in the mouse, with the qualifier doing real work. Human evidence remains observational, and Section 4.6 sets out what that evidence does and does not establish.

### 8.2 Study designs that would advance the field

Four designs follow from the four questions. Compartment-specific and inducible deletion, applied separately to endothelium, macrophage, and smooth muscle, and initiated at defined lesion stages, would separate the vectors that global knockouts confound and would settle whether the matrix arm survives the loss of the transport arm. Sex-stratified analysis should become routine rather than exceptional, given that oestrogen inhibits caveolar transcytosis and that recent lipid transport phenotypes are more pronounced in females. Single-cell and spatial profiling of human specimens, relating regional caveolar density to lipoprotein retention and to inflammatory state within the same plaque, would test whether the mouse network holds in human tissue. Finally, selective interference with the SR-B1 to DOCK4 interface, rather than with SR-B1 itself, would test the therapeutic hypothesis in the only form in which it is coherent. A staged research roadmap, mapping current mechanistic gaps to the experimental designs and translational milestones required to address them, is outlined in Figure 7.

**Figure 7:**
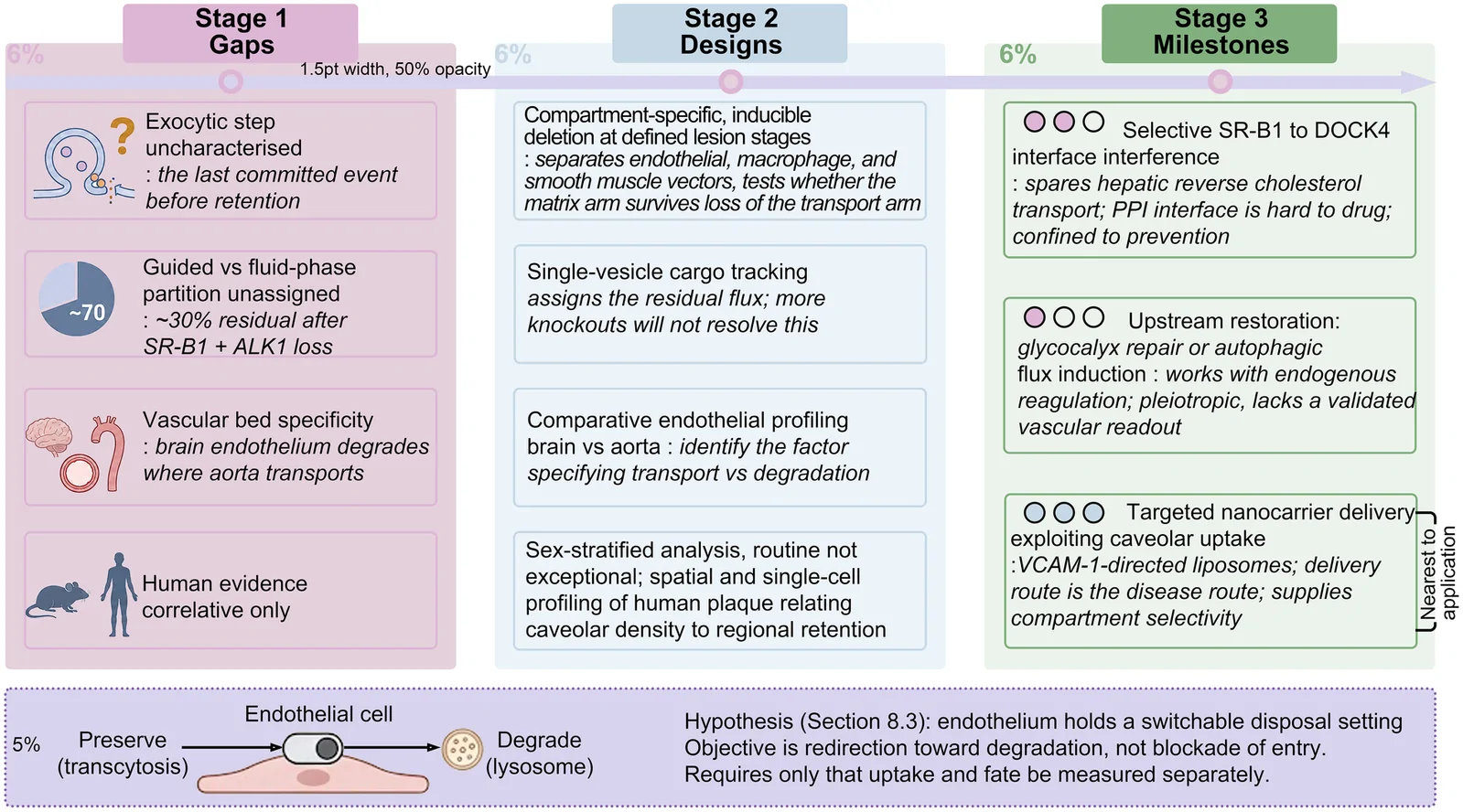
A staged roadmap from current mechanistic gaps to translational milestones. Proposed research trajectory. Stage one addresses the four principal gaps identified in this review, namely the uncharacterised exocytic step, the unassigned partition between receptor-guided and fluid-phase transport, the determinants of vascular bed specificity revealed by the degradative behaviour of brain endothelium, and the absence of causal human evidence. Stage two specifies the study designs that address them, comprising compartment-specific and inducible deletion initiated at defined lesion stages, single-vesicle cargo tracking, sex-stratified analysis, and spatial profiling of human specimens. Stage three sets the translational milestones, comprising selective interference with the scavenger receptor class B type 1 and dedicator of cytokinesis 4 interface, upstream restoration through glycocalyx repair or autophagy induction, and targeted nanocarrier delivery that exploits caveolar uptake rather than suppressing it. The dashed lane records the central hypothesis advanced in Section 8.3, that endothelial cells possess a switchable disposal setting and that redirection of lipoprotein toward degradation is a more achievable objective than blockade of entry.

### 8.3 A hypothesis worth testing

We close this section with a proposal that is speculative and, we believe, tractable. The observations that brain endothelium degrades rather than transports LDL, that autophagic flux governs Cav-1 abundance and thereby transport, and that the LDL receptor route consumes the particle while the SR-B1 and ALK1 route preserves it, together suggest that endothelial cells possess a switchable disposal setting rather than a fixed transport rate. On this reading, atherogenic endothelium is not simply endothelium with more caveolae; it is endothelium in which the balance between the consuming and the preserving route has shifted toward preservation. If that is right, the therapeutic objective is not to reduce lipoprotein uptake but to redirect it toward degradation, which is a different and considerably more achievable goal than blocking entry outright. Testing it requires only that uptake and fate be measured separately, which current methods permit and current studies rarely do.

## 9 Conclusions

Caveolin-1 occupies an unusual position in vascular biology. It is not a receptor, an enzyme, or a transcription factor, and yet it determines how much lipoprotein enters the arterial wall, how the endothelium reads its mechanical environment, and how the resident immune cells respond to what arrives. That breadth is why it has been difficult to characterise and why the literature about it appears more contradictory than it is.

The evidence supports several conclusions with reasonable confidence. Caveolae-dependent transcytosis is the principal route by which native lipoprotein crosses intact endothelium, and Cav-1 is required for it. The receptor logic is resolved in useful detail, with SR-B1 and ALK1 capturing the particle within the caveola and the SR-B1 to DOCK4 to RAC1 axis committing it to transit. Atheroprotection conferred by Cav-1 loss is not explained by nitric oxide and is better attributed to reduced transport and inflammation. Caveolar function is downstream of the glycocalyx and is set in part by autophagic turnover, which places Cav-1 within a control system that responds to both mechanical and metabolic input.

Equally, several conclusions are not yet available. The exocytic step remains uncharacterised. The partition between guided and fluid-phase transport is unassigned. The macrophage and endothelial pools act in opposite directions, and no existing intervention distinguishes them. Endothelial Cav-1 governs initiation but not progression, which confines the target to prevention unless the matrix arm proves separately addressable. Human data are correlative throughout.

The human layer deserves a sentence of its own, because it is thinner than the mechanistic material and points in two directions at once. Common variation at the CAV1 locus associates with coronary artery disease through higher CAV1 expression, which agrees with the endothelial transport account, whereas plaque and circulating Cav-1 are lower in established disease, which agrees with the macrophage and matrix account. Both can be true, and under the sign inversion argued here both are expected. No approved drug targets caveolar transport, although statins lower endothelial Cav-1 as a consequence of cholesterol depletion, PCSK9 inhibition alters the fate of the internalised particle, and SGLT2 inhibition plausibly raises Cav-1 turnover through AMPK. If a compartment-selective agent is ever built, it will be built as a delivery system rather than as a conventional small molecule, and the engineering requirement is now specific enough to state: an internalising address on activated arterial endothelium, and a payload chosen separately for each compartment.

The most useful reading, in our view, is that Cav-1 is a converter. It takes a physical input at the membrane, the flow pattern and the glycocalyx that reports it, together with a metabolic input, the nutrient state that sets autophagic flux, and it produces a transport rate that determines the substrate load of the intima and therefore the immunometabolic trajectory of the wall. Each arm of that statement is supported. The value of stating it as one sentence rather than four is that it identifies where intervention would be leveraged, namely at the conversion itself, and it explains why interventions applied to Cav-1 as a whole have not worked and are unlikely to. The field does not need more knockouts. It needs the ability to change what the endothelial cell does with a lipoprotein particle after it has bound it, and the brain endothelium demonstrates that such a setting exists.
